# The Geometry of Locomotive Behavioral States in *C. elegans*

**DOI:** 10.1371/journal.pone.0059865

**Published:** 2013-03-28

**Authors:** Thomas Gallagher, Theresa Bjorness, Robert Greene, Young-Jai You, Leon Avery

**Affiliations:** 1 Department of Biochemistry and Molecular Biology, Virginia Commonwealth University, Richmond, Virginia, United States of America; 2 Department of Psychiatry, University of Texas Southwestern Medical Center at Dallas, Dallas, Texas, United States of America; 3 Department of Physiology and Biophysics, Virginia Commonwealth University, Richmond, Virginia, United States of America; Harvard University, United States of America

## Abstract

We develop a new hidden Markov model-based method to analyze *C elegans* locomotive behavior and use this method to quantitatively characterize behavioral states. In agreement with previous work, we find states corresponding to roaming, dwelling, and quiescence. However, we also find evidence for a continuum of intermediate states. We suggest that roaming, dwelling, and quiescence may best be thought of as extremes which, mixed in any proportion, define the locomotive repertoire of *C elegans* foraging and feeding behavior.

## Introduction

Food is one of the most important determinants of an animal’s behavior. Some of the effects of food are obvious: if there is food, an animal may eat, while if there is no food, or if the food available is poor in quality, it may instead search for new food (see, e.g., Shtonda and Avery [1]). But other effects are complex and depend on the animal’s internal state: how recently it has eaten, the presence of food in the digestive tract, the quantity and nature of stored reserves such as fat or glycogen. Information about nutritional state is communicated within the animal by a complex and only partly understood system of signals, and much of the animal’s computational machinery is devoted to dealing with food and nutrition [2]. Better understanding of these signals might help in treating disorders of feeding, nutrition, and energy balance ranging from anorexia to obesity.

Despite its simple nervous system, the nematode *C elegans* has a complex array of signals to control feeding and food-related behavior [3]–[5]. Indeed, it is only a small oversimplification to say that in the *C elegans* hermaphrodite *all* behavior is food-related, since food and nutritional state affect every behavior that has been tested, often profoundly. Locomotive behavior has been studied with particular intensity. Previous workers have described three behavioral states that characterize the locomotive response to food: roaming, dwelling, and quiescence.

When actively feeding, worms alternate between roaming and dwelling [6]–[8]. Roaming worms move swiftly and relatively directly from one place to another, while dwelling worms move slowly and reverse frequently, thus covering little distance. Roaming and dwelling are respectively exploration and exploitation behaviors. Shtonda and Avery [1] and Ben Arous et al. [7] showed that worms roam more on low-quality food and dwell more on high-quality food. An additional behavioral state, quiescence, has recently been identified and characterized as a sleep-like state [9]–[11]. We found that worms enter quiescence when they become satiated [11]. Together, these studies show that locomotive activity is determined by nutritional status and that nutritional status can regulate switching between behavioral states.

We undertook the work described here to solve a particular problem: measuring satiety quiescence. Satiety quiescence has been particularly difficult to study, because quiescent worms are easily disturbed. In fact, it has not been possible to watch satiety-induced quiescence for more than about a minute, since for reasons that are not fully understood, quiescent worms wake up under continuous observation, even under conditions where they can be shown to spend most of their time quiescent when not observed [11], [12]. One consequence of this limitation is that we know little of the kinetics of quiescence: do worms cycle in and out of quiescence, and if so, at what rate? Which molecular mechanisms and which neurons and circuits regulate it? Our efforts succeeded: we can now measure satiety quiescence, and in future publications we hope to answer some of the mechanistic questions. However, in the course of this work we made an unexpected discovery, which is the focus of this paper.

We analyzed behavior using movement tracking and hidden Markov model analysis. Using this method we were able to identify behavioral states in recordings of movement that correspond to roaming, dwelling, and quiescence. However, the new method allowed us to describe behavior more precisely than previously, and as a result we could see something that was missed before. We found, to our surprise, that behavior seemed not to be limited to these three previously described states. A range of intermediate states also occurred. These states, taken together, suggest the existence of a behavioral state space with a triangular shape. The vertices of the triangle are pure roaming, dwelling, and quiescence, and the interior is occupied by mixed states. We suggest that roaming, dwelling, and quiescence are best thought of as archetypal states that can be mixed to form the range of locomotive foraging and feeding behaviors available to the worm.

## Results

### Roaming, Dwelling, and Quiescence can be Detected by HMM Analysis

#### Motion recording and analysis

To quantify quiescence over relatively long time periods, we developed an automated procedure to monitor worms. Because quiescence is suppressed by the presence of other worms [11], we recorded only a single worm at a time. To avoid mechanical disturbance, we did not mechanically track the worm, but instead placed it on a small spot of food, which did not move during recording. To test whether worms became quiescent under these conditions, we measured their speed of movement, assessing quiescence by counting time points at which speed was less than 1 µm s^−1^. Time at such low speeds was greater under conditions that promote quiescence, rising to 20% for worms fasted, then refed on good food (data not shown). These results suggest that satiety quiescence occurred under our recording conditions, although probably not at the level previously inferred for completely undisturbed animals [11].

**Figure 1:**
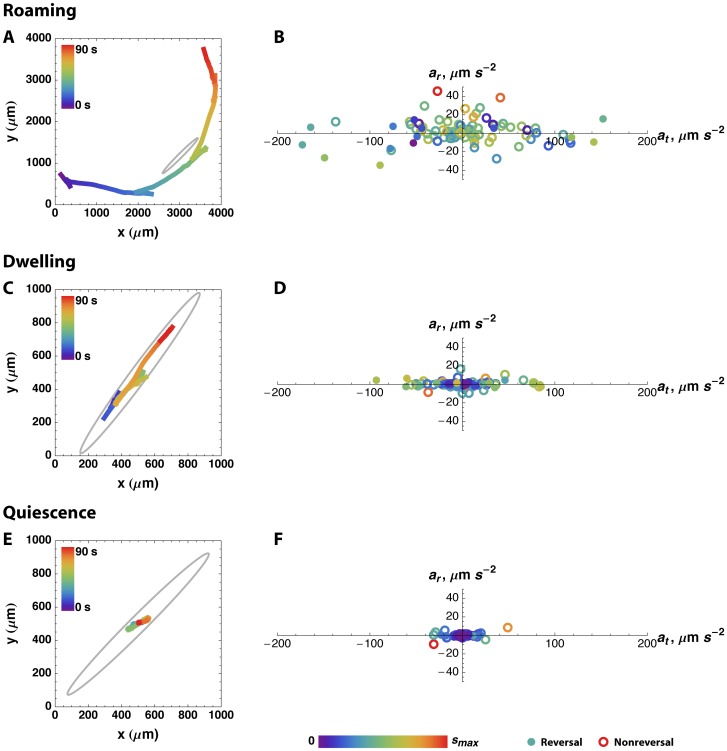
Motion characteristics of roaming, dwelling, and quiescence. Short movie segments illustrating statistically typical roaming (A, B), dwelling (C, D), or quiescence (E, F) (chosen as described under Statistically typical tracks in Methods) were analyzed to determine speed, acceleration, and reversal at each time. The tracks are shown in A, C, and E. Time is indicated by color. Note the difference in scale between A and the other two. The grey ellipses are 1.2 mm long×0.1 mm wide, about the size of the worm. B, D, F: Tangential and radial acceleration are plotted on the *x* and *y* axes. Speed is indicated by color, with the lowest and highest speeds indicated by purple and red. (Color is normalized within each track, so that, for instance, red points within the dwelling plot represent a lower speed than red points in the roaming track.) Reversal is indicated by filled circles, and nonreversal by empty circles.

Previous studies [6], [7] quantified two characteristics of the worm’s motion: speed and change of direction (referred to as “curvature” by Ben Arous et al. [7] and “turning” by Fujiwara et al. [6]). Change of direction cannot be measured accurately when the worm is moving slowly. To solve this problem, we measured speed, change of speed (tangential acceleration), reversal, and turning (radial acceleration) from each set of three successive points (see Motion characteristics in Methods). To illustrate motion characteristics of roaming, dwelling and quiescence, we show three short movie segments that illustrate typical roaming, dwelling, and quiescence behavior (Figure 1; see Statistically typical tracks in Methods). We found two differences between roaming and dwelling. First, consistent with Fujiwara et al. [6], reversals were much more frequent in dwelling. Second, during dwelling acceleration was correlated with speed. During roaming, in contrast, there was no obvious correlation of speed with acceleration.

Our results showed mostly low radial acceleration during dwelling, which appeared to contradict its previous description as the state with frequent changes in direction. However, after calculating speed and absolute angular change in direction across all our tracks, we found that change in direction is almost entirely reversal. “Change in direction” conflates two distinct behaviors, reversal and turning. The large average angles reported previously for dwelling and roaming [7] are because a majority of nonreversals–angles near 0°–are averaged with a substantial minority of reversals–angles near 180° (Figure S1, Figure S2).

#### Standard state fits

**Figure 2:**
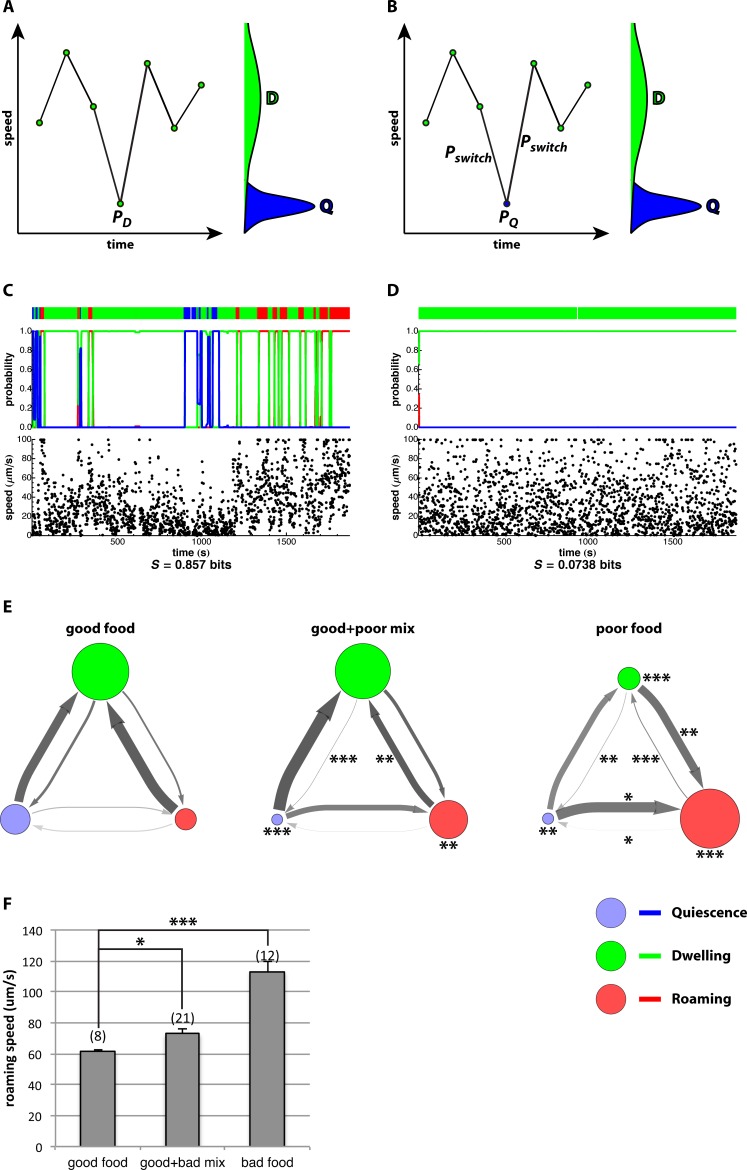
Hidden Markov model analysis, standard state fits. A, B. A simplified explanation of how HMM analysis uses both time and behavior to determine state. The plots show a hypothetical record of speed vs time. The bell-shaped green and blue curves at the right of each plot show the probability for a dwelling or a quiescent worm to move at a given speed. The distributions overlap, because while dwelling worms usually move faster than quiescent worms, at some time points they move as little as a quiescent worm. (Although a quiescent worm doesn’t move at all, its measured speed will usually be positive because of small errors in the measurement of its position.) The problem is to determine what state the worm was in at the central time point, where it did not move. Looking at this point alone, one would conclude that the worm was probably quiescent, because the probability for a quiescent worm to move so slowly (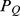; panel B) is much higher than the probability that a dwelling worm will do so (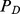; panel A). However, the behavior of the worm immediately before and immediately after is inconsistent with quiescence. Therefore, if the worm is quiescent at the central time point, it must have switched from dwelling to quiescence immediately before and must switch back immediately after. The probability that the worm is quiescent is therefore 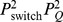. If the time between points is small, the probability of a switch, 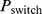, is a small number, and 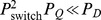. The worm is thus correctly inferred to be dwelling. The actual analysis is more complicated, since other motion characteristics than speed are used, and a probability is assigned to each state at each time point. C. The results of a standard state fit to a wild-type track. The lower plot shows speed; red, green, and blue lines in the upper plot show probability of the roaming, dwelling, and quiescence state at each point in time. The color bar at the top summarizes the probabilities. (The small gap is a brief period of missing data.) The change in behavior with time is most easily seen by looking at the frequency of very low speed (<20 µm/s). Such time points are a majority in quiescence, a substantial minority in dwelling, and almost absent in roaming. Most time points are assigned to a single state with near 100% probability, and the worm spent a substantial amount of time in each of the three. This is reflected in the high excess entropy, 0.857 bits. D. The results of a similar fit to the same data as in C, but scrambled into random order. The three-state fit did not have substantially more information than a single behavioral state, as shown by the very low entropy (*S*). E. Rate graphs summarizing state probabilities and transition rates between states based on analysis of well-fed wild-type worms on either good food (*E coli* HB101), poor food (HB101 treated with aztreonam) or a mixture of good and bad. The area of each circle is proportional to the amount of time worms spend in that state (red = roaming, green = dwelling, blue = quiescence). Thicker arrows represent faster switching from one state to another. Darker arrows are more accurately measured, lighter grays represent less accurate measurements, based on variability from one worm to another. *P<0.05, **P<0.01, ***P<0.001, different from good food, Mann-Whitney *U*-test. Thus, for instance, worms switch from dwelling to roaming more rapidly (*P*<0.01) on poor food than on good and spend more time roaming (*P*<0.001). Number of worms for each graph as in F. Dataset S1 contains the raw data on which these rate graphs are based for all experiments in this work. F. Mean speed of roaming worms. These data are based on the same tracks as E. Number of worms in each experiment is shown above the bar. *P<0.05, ***P<0.001, Mann-Whitney *U*-test.

To capture the information available in the time course of behavior, we used a hidden Markov model (HMM). Behavioral state can’t be reliably determined by looking at a single point in time. For instance, although a dwelling worm moves most of the time, there are time points at which no detectable movement occurs. By themselves, these cannot be distinguished from quiescence. However, this ambiguity can be resolved by looking at the time course of behavior. A dwelling worm is still only at isolated points in time, while a quiescent worm remains so almost continuously. In HMM analysis the state inferred at one time depends, not just on behavior at that time, but also on states immediately before and after (Figure 2A, B).

We deduced the characteristic behavior of roaming, dwelling, and quiescent worms from records acquired under conditions in which worms have been reported to spend most of their time in just one of these states (see Standard state fits in Methods). Figure 2C shows the result of such a fit to a recording of a well-fed wild-type worm on good food. Although there were brief periods during which behavior was ambiguous (e.g., just before 1000 s, when there is a ∼75% probability of dwelling and ∼25% of quiescence), at most times one state was identified with close to 100% confidence.

We developed a statistic, excess entropy, to quantify the extent to which the analysis helped to explain behavior. The fit in Figure 2C had an entropy of 0.86 bits. (The maximum possible is 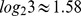.) To test if the fit truly detected coherent time-dependent changes in behavior, we scrambled the data and repeated the fit. Figure 2D shows an example of one such fit to scrambled data. No state changes are detected, and the entropy is only 0.074 bits. Fits of 363 recordings from 49 experiments (Table S1) had entropies of 0.65±0.17 (mean ± standard deviation; range 10^−6^–0.98). In contrast, fits to 7260 scrambled records had entropies 0.13±0.13 (3×10^−10^–0.67). The difference between these distributions is significant (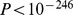, Kolmogorov-Smirnov test).

Using this analysis, we confirmed and extended earlier results. For instance, low-quality food suppresses quiescence [11] and promotes roaming [1], [7]. We confirmed these results (Figure 2E). Further, our analysis allowed us to estimate the rate at which worms switch from one state to another. The suppression of quiescence was explained mainly by a decrease in the rate at which worms switch from dwelling to quiescence (Figure 2E).

### The Behavior of a Roaming Worm Varies Depending on History, Food Quality, and Genotype

A simple hypothesis for the control of locomotory behavior is that food quality and other conditions affect only the rates at which worms switch between states. Under this hypothesis worms on poor food would spend more time roaming, but during the time they spend roaming, worms would behave the same on good food and on poor food. The alternative is that the behavior of a worm depends not only on the state it is in, but also on conditions. Under this hypothesis roaming worms might behave differently on good food and on poor food.

To test these hypotheses, we compared the motions of worms in the same state under different conditions. Figure 2F shows an example: the speed of worms on good food, poor food, or a mixture, measured only during the time they spent roaming. The simple hypothesis was decisively rejected. Roaming worms on poor food moved faster than roaming worms on good food and roaming worms on mixed food. (Ben Arous et al. [7] also reported that roaming worms move faster on poor food.) The conclusion generalized to motion characteristics other than speed and states other than roaming. Dwelling worms moved differently depending on conditions, and there was even a suggestion of changes in the small restless movements that sometimes occur during quiescence (data not shown).

### Unbiased State Discovery

The observation that the behavior of a roaming worm depends on conditions such as food quality raised a difficult question: how are roaming, dwelling, and quiescence defined? Above we claimed that roaming worms moved faster on poor food. This claim is correct, if roaming is defined by the motions of worms under conditions that have been reported to promote roaming. However, speed is one of the characteristics that distinguishes dwelling and roaming. If poor food caused dwelling worms to move faster, they might be classified as roaming. If poor food in addition caused dwelling worms to reverse less and to accelerate less, any method that deduces behavioral state from these characteristic motions would classify the behavior as roaming.

**Figure 3:**
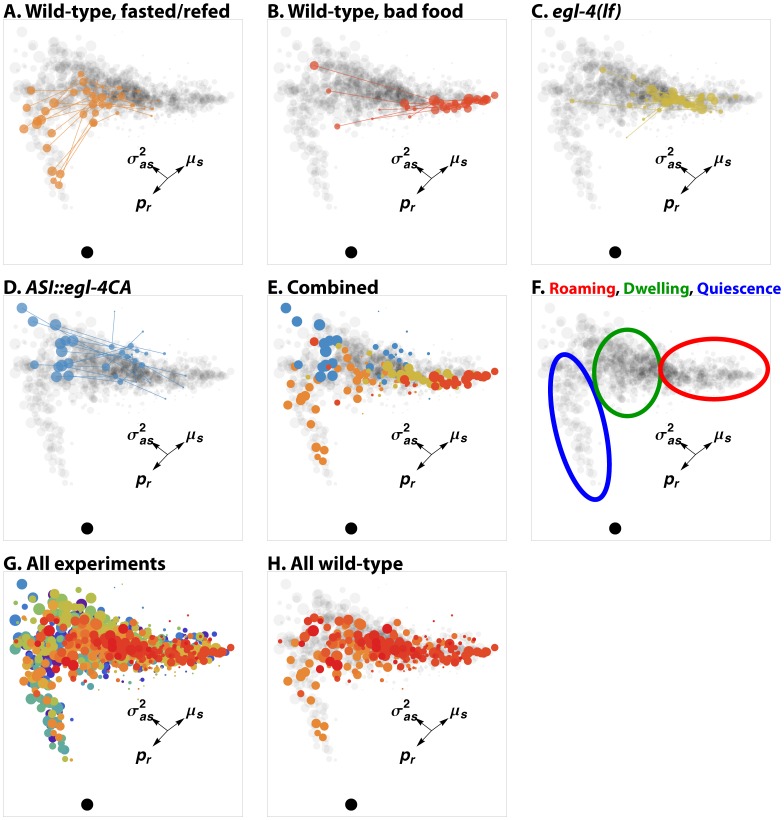
Geometry of behavioral states. This figure shows the two-dimensional arrangement of behavioral states discovered by unbiased open-loop fits. Each circle (except the black one near the bottom of each panel, which represents complete immobility) represents a single state from a single worm. The area of the circle is proportional to the amount of time the worm spent in that state. The gray background in A–F and H, representing all states discovered in all experiments, is shown for context. States are colored by experiment; the same colors are used in panels A–E and G–H and in Figure S5. Arrows show the directions in which three of the seven state parameters increase. 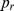 is the probability of reversal, 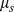 is mean deskewed speed, and 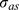 is the covariance of deskewed speed and acceleration. A–D: States discovered in four experiments. Lines join states discovered in the same worm. A. 14 wild-type worms, fasted for 12 hours, refed on good food (*E coli* HB101) for 3 hours, then recorded on good food. B. 12 wild-type worms, grown on good food and recorded on poor food. (Poor food is HB101 treated with aztreonam, which prevents cell division [7].) C. 12 mutant worms lacking cGMP-dependent protein kinase (PKG, encoded in *C elegans* by *egl-4* [6]), grown and recorded on good food. D. 12 transgenic worms that express constitutively active PKG in ASI neurons, grown and recorded on good food. E. States from the four previous experiments plotted together. F. Regions of the triangle can be identified as roughly corresponding to roaming, dwelling, and quiescence, as described in the text. G. All behavioral states discovered in 49 experiments on 363 worms. H. States from all experiments on wild-type worms (80 worms total). These experiments differ only in whether the worms were well-fed or starved and refed, and in the quality of food on which they were recorded.

To address this problem, we developed an unbiased analysis in which state characteristics are derived directly from the behavior of a single worm (Figure S3; see Unbiased closed-loop fits in Materials and methods). Our fits of 363 recordings yielded a total of 1083 state descriptions from 357 three-state and 6 two-state fits. A state description is the list of seven parameters that specify such behavioral characteristics as the probability of reversal, the mean speed, and the correlation between speed and acceleration. Each state is thus a point in a seven-dimensional space. Interestingly, however, most of the points lay close to a plane–93% of the variance is captured in two dimensions. It was thus possible to plot them in two dimensions while preserving most of their geometric relationships. Figure 3 shows such plots.

We were able to identify regions of the plot that correspond to roaming, dwelling, and quiescence by considering their motion characteristics and by comparing our results with published results. The arrangement of states is roughly triangular (Figure 3G). The location corresponding to immobility is near the lower left, so this is the direction of quiescence. Speed increases towards the upper right of the plot, while reversal increases towards the lower left. Thus, upper right is the direction of roaming, which is characterized by high speed with few reversals. Covariance of acceleration and speed increases towards the upper left, which is thus the direction of dwelling.

To more precisely identify regions with states, we looked at the results of specific experiments. Wild-type worms fasted for twelve hours then refed with good food for three hours alternate between quiescence and dwelling [11]. (When not observed, worms so prepared spend most of their time quiescent, but watching them disturbs them in some unknown way, causing them to wake and dwell [11]. Our recording conditions allowed some quiescence, but were disturbing enough that the worms also dwelled.) Each such worm had two high-probability states, one in a region close to the lower half of the left side of the triangle, and another near the center (Figure 3A), which we thus identified as quiescence and dwelling, respectively. On poor food wild-type worms roam. They spent most of their time in states near the right vertex (Figure 3B). The states of *egl-4(lf)* mutant worms, which spend most of their time roaming even on good food [1], were in the same general region (Figure 3C). Worms engineered to express constitutively active cGMP-dependent protein kinase in ASI neurons showed an unusual pattern that was never seen in wild-type worms. They alternated between two states, a less probable one near the boundary between dwelling and roaming, and a more probable one near the upper left corner of the triangle. We call the latter state hyperdwelling, since it exhibits the characteristics of dwelling even more strongly than a dwelling wild-type worm. Figure 3F summarizes the regions corresponding to roaming, dwelling, and quiescence.

### Are there Discrete Locomotive Behavioral States?

We were surprised that we did not find discrete, well-separated clusters corresponding to roaming, dwelling, and quiescence. Rather, as shown in Figure 3G, the observed states filled most of the triangle, sparing only the region between quiescence and roaming. This suggests that our previous view, that the worm has available to it three distinct patterns of locomotive behavior, might be too simple. Instead the worm may be able to continuously tune its behavior between these three patterns.

We considered three alternative explanations for the failure to observe discrete clusters of states. First, the clusters might exist but be blurred by noise. There is error in every measurement. Perhaps the errors were so great as to spread the clusters until they merged with each other, giving a false appearance of continuity. This explanation was refuted by looking at single experiments. Figure 3A–D clearly show well-defined clusters of states. Each of A and D, in fact, shows two well-separated clusters, and each worm in those experiments alternated between a state in one cluster and a state in the other. We clearly had the ability to resolve distinct patterns of behavior. Figure 3E emphasizes this by showing that the states discovered in the experiments of A–D occupy six distinct, well-defined positions.

Figure 3E suggests a second possible explanation for the lack of clusters. Although 9 of our 49 experiments were done on wild-type worms, the rest were done on various mutant genotypes. Perhaps normal worms do have discrete roaming, dwelling, and quiescence states, but the unnatural behavioral patterns of mutants fill up the blank regions between the wild-type states. In fact, it was obvious that without the *ASI::egl-4CA* and *egl-4(lf)* experiments, the wild-type states of Figure 3A,B would form three discrete clusters (red and orange states in Figure 3E). To test this, we plotted all the states discovered in experiments on wild-type worms (Figure 3H). Even when we looked only at wild-type, discrete clusters were not evident.

A third possible explanation for our failure to identify clusters is more complicated. The plots in Figure 3 show the disposition of states in two dimensions, but the actual state space is seven-dimensional. Perhaps roaming, dwelling, and quiescence are separated from each other in the full seven-dimensional space, but this separation is lost when they are projected onto a plane. While we cannot entirely exclude this possibility, we found no evidence for it. It is somewhat implausible on its face, since the two dimensions plotted capture 93% of the variance–any additional separation could occur only in the remaining 7%. We examined state plots in 3 dimensions and looked at projections onto planes containing each of the seven dimensions and found no evidence of discrete clusters. In addition, we attempted to automate the search for clusters using hierarchical cluster analysis based on all seven state characteristics (Figure S4). The results were disappointing. While by design cluster analysis always finds clusters, the state clusters were excessively sensitive to the details of the algorithm (different distance measures and linkage methods often produced widely different clusters) and to the data included (during the course of this work clusters often changed radically with the addition of a few new recordings). Furthermore, the clusters failed basic experimental consistency criteria. For instance, if the red, green, and blue clusters in Figure S4 corresponded to roaming, dwelling, and quiescence, we would expect that fasted and refed wild-type worms would alternate between a blue state and a green state. Some of them did, but in others the two main states were both green. We do not believe that the clusters identified by cluster analysis have any biological reality.

### Behavioral States are Arranged in a Triangle

**Figure 4:**
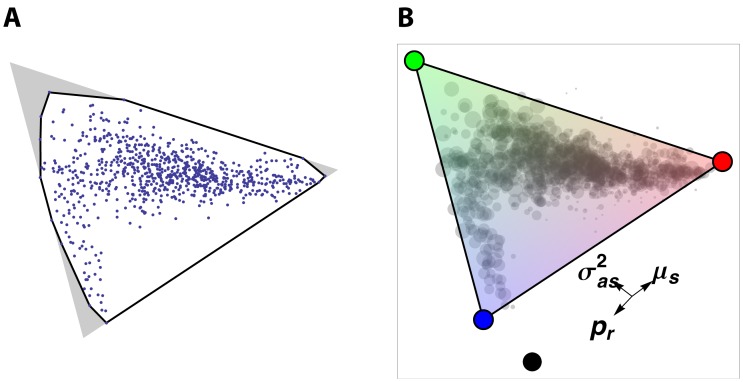
Behavioral states are arranged in a triangle. A. Each of the 832 states with probability greater than 10% is plotted in two dimensions as in Figure 3. The black line is the smallest polygon that contains all of them (the convex hull). The area of this polygon is 90.5% that of the smallest triangle containing them, significantly greater than that expected if they are not constrained to a triangle (*P*<10^−5^). The corresponding figure for a test using all the states, not just those with probability greater than 10%, is 90.8% (*P*<10^−5^). B. An interpretation of the triangular state space. We suggest that the locomotive behavioral patterns available to a worm can be any mixture of three archetypal patterns, represented as red, green, and blue circles. Like primary colors, these mix to form a triangle of possibilities.

Looking at the arrangement of all states (Figure 3G), we were struck by the impression that they fill out most of a triangle. To test this impression, we used a test recently described by Shoval et al. [13]. We compared the area of the smallest polygon that contains the states to that of the smallest triangle that contains them (Figure 4A). If they were really arranged in a triangle, the smallest polygon that contains them would be a triangle and the ratio of areas 1. Non-triangular points, in contrast, would give a smaller ratio. (For a circle, for instance, the ratio is ∼0.605.) The actual ratio, 0.916, was significantly greater than that expected for a random arrangement of points at 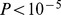.

### Change of Behavior with Time

The discovery of a continuous state space raises the possibility that a single worm can vary its behavioral state continuously in time. For instance, behavior depends on food quality and feeding history (Figure 1F, Figure 3A, B, H). This suggests that a worm that sees a change in the quality or quantity of its food might respond by gradually changing its behavior.

To test this hypothesis, we developed a way to measure how similar the behavior in one recording is to another (see Fit and state dissimilarity in Methods). To validate this measure, we first tested the hypothesis that identically treated worms of the same genotype would behave similarly to each other. The dissimilarity of tracks within the 49 experiments was 0.60±0.60 (mean ± standard deviation; range 0.016–5.3); between experiments it was 1.41±1.52 (range −0.0007–12). The difference between the two dissimilarity distributions was significant (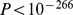, Kolmogorov-Smirnov test). Figure S5 shows the clustering of tracks within experiments graphically.

The experiments discussed above were designed to produce stable behavior: the worms were given a long time to adapt to conditions, and recordings lasted at most an hour. To see how behavior changes with time, we placed starved worms on food and recorded their behavior for four hours, starting immediately, then broke each track into 15 min segments for analysis (Figure S6).

**Figure 5:**
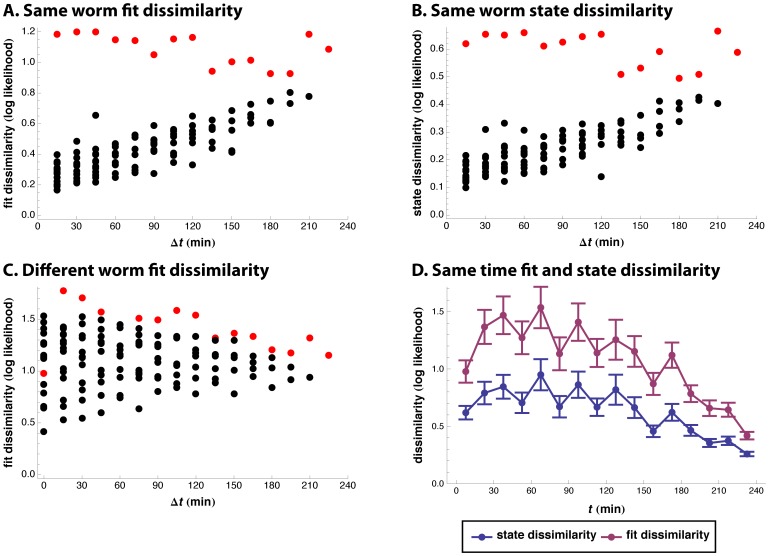
Behavior change during recovery from starvation. A. Each point is a mean of fit dissimilarity over 14 worms recovering from starvation. The dissimilarities are between two 15 min cuts recorded from the same worm, and they are plotted against the time difference between the cuts. For instance, one of the points at 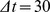 averages the dissimilarity between the 15–30 min and the 45–60 min cuts of worm 1, the dissimilarity between the 15–30 min and the 45–60 min cuts of worm 2, …, and the dissimilarity between the 15–30 min and the 45–60 min cuts of worm 14. Other points at 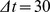 average dissimilarities between 30–45 min and 60–75 min cuts, between 45–60 min and 75–90 min cuts, …, and between 195–210 and 225–240 min cuts. Dissimilarities involving 0–15 min cuts are highlighted in red, e.g. the red point at 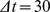 averages dissimilarities between the 0–15 min and the 30–45 min cuts. The 0–15 min behavior was very different from behavior at all later times. Aside from this exception, a given worm’s behavior changed only gradually with time, as shown by the gradual increase in dissimilarity with time interval. B. Like A, except that state dissimilarity is plotted instead of fit dissimilarity. C. Individual worms behave differently from each other. As in A, each point is an average of fit dissimilarities between cuts separated by 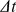 in time, but here each worm is compared not to itself, but to other worms. D. This plot shows mean ± standard error of between-worm dissimilarities plotted against time. The points of the fit dissimilarity plot are the same as those at 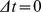 in C, but now plotted against the time at which they were recorded. Both fit and state dissimilarities start out high, but decrease with time as the worms settle into their new behavior.

If a worm’s behavior changes gradually with time, its behavior should be more similar at short time intervals than at long. Figure 5A shows that this was the case. Behavior during the first 15 min was very different from all subsequent times (probably because of the enhanced slowing effect [14]) as shown by the red points. But, excluding this segment, a worm’s behavior changed gradually with time interval. (Even including the red points, the association between dissimilarity and time difference is significant at 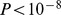; see Starvation recovery statistical tests in Methods.) A gradual change in behavior need not imply that states change continuously–it is possible that the states remain the same, but their probabilities change gradually. In an attempt to test this, we developed an alternative measure, state dissimilarity, in which only state characteristics are used in the comparison of behavior, not state probabilities or transition frequencies. By this measure as well behavior changes gradually with time interval (Figure 5B; 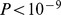). This suggests that the states of a single worm are not discrete, but can change continuously. This is only a suggestion, however, since state dissimilarity may not be completely immune to effects of state probabilities.

The gradual increase in dissimilarity with time interval was not visible in comparisons between different worms. Different worms were no more alike at the same time than after intervals of hours (Figure 5C; 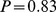). This shows that behavior varies from one worm to another–a worm is more like itself 30 min later than like a genetically identical worm with identical history. While there is nothing fundamentally shocking about this discovery, it surprised us, because the different-worm dissimilarities were roughly twice as large as the same-experiment dissimilarities measured previously. The contradiction was resolved by looking at how behavior varied with time. Both fit and state dissimilarities between worms began high, but decreased with time (Figure 5D; 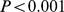). After four hours mean fit dissimilarity was 0.42, comparable to the mean within-experiment dissimilarity of 0.60 measured above. This suggests that the worms reacted diversely to a sudden change in the environment, but settled into similar behavior with time. State dissimilarity showed the same pattern.

## Discussion

Behavioral states like sleep and hunger reflect patterns of excitable cell activity. For instance, in humans sleep is associated with certain types of activity or inactivity in the thalamus. Questions about the nature and relationships of behavioral states are interesting because they give us hints of the underlying patterns of neural activity. Discrete states, for instance, might suggest the existence of one or a small number of on-or-off neurons. Continuous states would be difficult to reconcile with such a mechanism. Furthermore, to investigate the neural basis of behavioral states, for instance by testing the effect of activating specific neurons or imaging neural activity in a worm that switches state, we need to be able to measure states.

### Hidden Markov Models

HMM analysis improves on previous methods of analyzing *C elegans* locomotion. Behavior is inherently variable, and as a result it is difficult to identify the type of behavior an animal is engaged in by looking at a single point in time. HMM analysis is a simple way of using the information present in the time course of behavior to mitigate this problem. Markov models have previously been used to characterize behavioral sequences (e.g. [15], [16]). Their usefulness does not depend on the (highly implausible) assumption that the animal under study actually is a Markov machine. The Markov model is useful because it makes minimal assumptions about the timing of state transitions. The inferred behavior is therefore driven by the data, rather than being determined by the model. HMM analysis has been used to analyze behavior in *C elegans* [17], mice [18], and humans [19]. HMM analysis allows the use of Markov models with noisy data, can be applied to multidimensional data series, and, as we have shown, allows automation of the recognition of behavioral patterns.

### Continuous State Space

Based on previous results, we expected to find three distinct and well-separated patterns of behavior corresponding to roaming, dwelling, and quiescence. These would appear as three clusters of states. While clusters would not be perfectly tight (it had already been reported, for instance, that the speed of roaming worms depended on food quality [7]), we expected that states within a cluster would be far more similar to each other than to states in another cluster. Such clusters can indeed be found in many individual experiments (e.g. Figure 3A, D). But taking all experiments as a whole, or even experiments on wild-type, no discrete clusters are evident (Figure 3G, H).

Although this result surprised us, it is entirely consistent with past results, if not necessarily with published interpretations of those results. The hypothesis that the worm switches between two or three discrete states is based mainly on two observations: that the pattern of behavior shows large changes with time, and that these large changes in behavior are often abrupt. In fact, we also see large abrupt changes in behavior in our recordings. Discrete state models do indeed imply abrupt changes in behavior. But while continuous state models do not necessarily imply abrupt changes in behavior, they are consistent with them.

It is not surprising that past workers missed these intermediate behavioral states, for two reasons. The first is simple: they may not have been looking for them. For instance, in our work on satiety quiescence [11], we were not trying to find behavioral states, but only to distinguish whether a worm was quiescent or not. The other reason is the technical limitations of past analyses. First, deskewing is important. Without this transformation, everything that happens at low speed is compressed into a small region of behavioral space, and it becomes difficult to make any distinctions other than fast movement and slow movement. Second, the past analyses didn’t distinguish enough behavioral dimensions. Both Fujiwara et al. [6] and Ben Arous et al. [7] were aware of this problem and attempted to solve it by measuring two motion characteristics: speed and change of direction. However, our results show (and this is consistent with data in both papers) that change of direction is actually almost entirely reversal (Figure S2), and that probability of reversal is tightly inversely correlated with mean speed (Figure 3), although the tight correlation was obscured in past work by the noisiness of the change of direction measurement at low speed. Thus, Fujiwara et al. [6] and Ben Arous et al. [7] effectively measured only one behavioral dimension. Our analysis extracts four motion characteristics, reversal, speed, tangential acceleration, and radial acceleration, from the record. HMM analysis allowed us to measure many more derived characteristics, three of which we used: variance of speed, variance of acceleration, and covariance of speed and acceleration. Although there are correlations among these seven, behavior is still spread over two dimensions, and this is enough to separate intermediate behaviors.

### Pareto Optimization

Figure 4B shows an interpretation of the proposed continuous state space. We suppose that the worm has available to it three extreme patterns of behavior, shown as red, green, and blue circles. However, the worm can also mix these in any proportions, the way an artist mixes colors in a palette, to form intermediate patterns.

Recently, Shoval et al. [13] showed that the phenotypes of organisms trading off the abilities to optimally carry out three tasks can form a triangular pattern. The vertexes of the triangle are archetypes: phenotypes that are optimal for a single task. The optimum for an animal that needs to be able to execute two of the three will be on the line joining the two corresponding archetypes, and to execute all three in the interior of the triangle, at a point determined by the relative importance of the three tasks. In economics, the problem of finding such an optimum is called Pareto optimization. Pareto optimization has another consequence, which we also observe: dimensional reduction. The phenotypic space resulting from trade-offs between three goals is two-dimensional, even if the space of all possible (non-optimal) phenotypes has more dimensions. Although Shoval et al. [13] discussed the phenotypes of different species in a taxonomic group, their reasoning is equally applicable to the selection of patterns of behavior by an animal.

We suggest that the two-dimensional, triangular locomotive state space we discovered is explained by the need to trade off three goals. The archetypal roaming state, represented by the red circle in Figure 4B, is behavior optimized for finding new food. The archetypal dwelling state (green circle) is optimal for exploiting a food source, once it has been found. And the archetypal quiescent state (blue circle) is optimal for minimizing movement. The gap along the lower edge of the triangle also finds an easy explanation. It is there because no circumstances exist (or, at least, none were tested in our experiments) in which an animal tries to simultaneously satisfy the goals of remaining immobile and searching for food.

### Are there Discrete States?

Our results are consistent with the hypothesis that the time course of behavior of a single worm can be explained by discrete states. The discovery of a continuous state space would not directly contradict this hypothesis–it could be that continuity is only evident when one looks across many worms under a wide variety of conditions. It does, however, raise the possibility–conceivably even a single worm varies its behavioral states continuously in time. For instance, the behavior of a wild-type worm varied depending on food quality and feeding history (Figure 2F, Figure 3A, B, H). This implies that a worm that sees a change in the quantity or quality of its food will respond by changing its patterns of behavior. Since the detection of food and response to this change cannot be instantaneous, this suggested that a single worm might show a gradual change in behavior. We directly tested this possibility, and found that it was true (Figure 5). Furthermore, the results were consistent with continuous change in the states themselves, not just in the kinetics of switches among them.

Continuous states complicate our view of behavior. A simple version of the discrete state model that has until now implicitly guided the analysis of worm locomotion has three characteristics: (1) States are well separated from each other. (2) The switch between states is abrupt. (3) The characteristics of a state do not change with time. The discovery that the state space may be continuous suggests that all or some subset of these statements may be false. Our current analysis cannot test them, because they are embedded in the model on which it is based: a discrete-state Markov chain. We are working to develop continuous-state hidden Markov models that will allow us to separately test the three.

In addition to improved analysis, better data would help in determining whether there are discrete states, specifically in testing criterion 1. Our failure to see well-separated clusters of states may result in part from missing information. Currently we use only the average position of the worm, a single *x*, *y* point at each time. Two other types of behavioral information might be helpful: the shape of the worm [20], and feeding. Casual observations suggest that roaming, dwelling, and quiescent worms have distinct postures (our unpublished observations), and feeding stops during quiescence [11]. It is possible that these measurements would separate the state continuum into discrete clusters. Ultimately behavioral states must reflect the state of the nervous system. The most informative additional data would be the time course of activity of neurons in freely moving animals. Such experiments are challenging, but are being done [21]–[23].

## Materials and Methods

### Preparation of Bacteria

Five ml LB was inoculated with a single colony of *E. coli* strain HB101 expressing mCherry and incubated shaking overnight at 37°C. The culture was removed from the incubator and allowed to sit at room temperature overnight. The sample was centrifuged at 4,000 RPM for three minutes. After decanting the supernatant, the pellet was resuspended in the small residual amount of broth and transferred to a microcentrifuge tube. 40 µl of this suspension was twice serially diluted 1∶1 with M9 (for a final 4× dilution). 5 µl of this suspension was pipetted onto a 35 mm NGM plate and allowed to dry completely.

Aztreonam was used to prepare poor (i.e., low-quality) food [7]. Aztreonam prevents bacterial cell division, so that the bacteria turn into long snakes, which are difficult for the worm to eat. Aztreonam-treated bacteria were prepared as above with one additional step. After shaking overnight at 37°C, 1 mL of turbid LB was added to 4 mL fresh LB and aztreonam (Sigma-Aldrich) was added to a final concentration of 5 µg/mL. This was incubated overnight shaking at 37°C, and then allowed to sit at room temperature overnight.

### Locomotion Assays

L4 worms were picked to an HB101 seeded NGMSR plate and given 8 hours to develop to young adult stage. For fasted conditions, young adult worms (adults containing no eggs) were picked to individual 60 mm NGMSR plates without food and starved for 12–14 hours. A single starved worm was then transferred to a 6 mm diameter spot of bacteria made by placing 5 µl bacterial culture on a plate, focused under the camera, and allowed to refeed for 3 hours. The microscope light was then turned on and video capture was started at 1 frame/second for 1 hour. In the starvation recovery experiment the worms were treated identically, except that recording started immediately after transferring the worm to food and continued for four hours.

For nonfasted assays, worms were prepared identically except that young adults were transferred to a 60 mm NGM plate with food for 12–14 hours and worms were given 30 minutes on the assay plate to recover from being transferred, followed by taking a 30 minute video at 1 frame/second.

In the 49 experiments listed in Table S1, worms were recorded using a Leica MZ6 microscope at 2.5× magnification with a 1.0× lens and a Retiga-4000R camera and Image Pro Plus 6.2. These videos were analyzed by Image Pro Plus software. In the starvation recovery experiment, recordings were made on a modified version of the nine-worm recording station described by Shtonda and Avery [1] in which the worms were imaged through Computar MLM3X-MP macro zoom lenses onto Pointgrey GRAS-14S5M-C digital cameras, and the videos were analyzed by MATLAB scripts of our design. In all cases a low pass filter was applied to each frame of the movie and the light/dark threshold was adjusted to find the outline of the worm. The center of mass was calculated at each time, reducing each recording to a series of 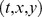 points, which were the basis for all subsequent analyses. In the starvation recovery experiment, each 4 hour track was broken up into 16 segments, each 15 min long.

A certain amount of motion is detected even from a completely stationary worm, as small fluctuations in measured brightness of border pixels cause them to vary above and below threshold. This noise motion places a limit on our ability to detect immobility and therefore quiescence. To quantify it, we recorded a worm immobilized with 30 µl of 1 M sodium azide before transfer to the assay plate. The mean speed of an immobilized worm was 0.32 µm s^−1^, and the speed was below 1 µm s^−1^ 99.7% of the time. Apparent motion was biased along one direction, as expected, since most border pixels are farther from the center in the anterior/posterior direction than in the dorsal/ventral direction.

### Motion Characteristics

#### Explanation

Fujiwara et al. [6] and Ben Arous et al. [7] both began their analysis by measuring speed and change of direction (called “turning” by Fujiwara et al. [6] and “curvature” by Ben Arous et al. [7]). Change of direction is a problematic measurement. From 3 consecutive center of mass positions 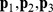 one calculates 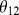, the direction from 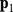 to 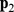, 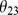, the direction from 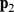 to 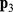, and finally the minimum angular difference required to get from heading 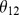 to 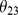. If the worm didn’t move between time 1 and time 2, 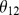 is undefined; if the worm moved little, 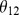 is measured with poor accuracy. Since change of direction is the difference between two angles, it is poorly determined if there is little movement between time 1 and time 2 or between time 2 and time 3. The fundamental problem is that the function mapping positions to change of direction is discontinuous at 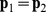 and at 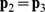. Since we needed to analyze behavior under conditions where the worms spend much of their time not moving, this was a problem. A less important problem is that speed and change of direction do not capture all the useful information in three consecutive points.

To solve these problems, we systematically redesigned the process of motion reduction, by which we mean the derivation of rotation and translation-invariant descriptions of motion from laboratory-frame Cartesian data. Three consecutive 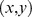 center of mass positions have six degrees of freedom. However, three of those describe where the petri plate is located and how it is oriented with respect to the camera and are of no interest in understanding the worm’s behavior. This leaves three numbers’ worth of useful information. A natural way to remove the translational information is to measure velocity, the change in position, and acceleration, the change in velocity. Each of these is a two-vector, and together they capture all the translation-invariant information. Furthermore, they are continuous functions of the positions, so they are well-defined for both small and large motions.

Removing the rotational degree of freedom is more difficult. One way to do this is to rotate the velocity and acceleration vectors into a coordinate system whose axes are defined by 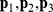 in such a way as to covary with the orientation of the petri plate. An obvious choice for the *x* axis of this coordinate system is the direction of the velocity vector. When 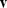 is rotated to point along the *x* axis, its *x* component is just the speed, and its *y* component becomes 0 and can be discarded. Speed is a continuous function of the positions. The acceleration vector 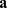, rotated into the same coordinate system, has two components, tangential acceleration 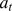, acceleration along the direction of motion, which measures the rate of change of speed, and radial acceleration 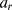, acceleration perpendicular to the direction of motion, which is proportional to the rate of change of direction.

Unfortunately, acceleration thus defined is *not* a continuous functions of the positions. The rotation to the direction of motion depends on accurate measurement of that direction, which depends on the worm moving. We first attempted to solve the problem by using the sum of two velocities 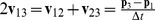 to define direction. This works well if the worm moves very little in the first time interval, the second time interval, or even in both time intervals. (In the last case, although the direction of the velocity vector is poorly defined, the acceleration is close to 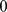, so the function is still continuous.) It is discontinuous only when the worm moves during the first time interval then reverses its movement during the second time interval, so that it ends up where it started. In practice, the worm need not reverse its motion exactly for this to be a problem–if the distance from 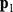 to 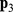 is close to or smaller than the typical position measurement error, acceleration is poorly defined. Although this does not occur frequently, it is common enough to be a problem, especially during dwelling.

Fortunately, when the worm reverses its motion another well-defined direction can be derived from the positions: the difference in velocities 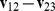. By itself the difference is no better than the sum, but by choosing the larger of the two in every case, we always have a well-defined direction vector if the worm moves at all. Furthermore, if acceleration is defined (conventionally) based on the difference of the two velocities when 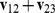 is larger and (unconventionally) on the sum of the two velocities when 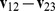 is larger, acceleration is a continuous (though not differentiable) function of the positions.

There is one lingering problem: one bit of information has been lost. From the number 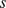 and vector 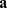 we cannot tell which branch of the calculation was followed. For instance, 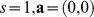 is consistent both with constant movement in one direction and back-and-forth movement. To preserve this bit of information, we defined a new motion characteristic, 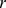 for reversal, which is 1 when 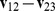 is larger and 0 when 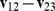 is larger. (Equivalently, 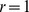 if and only if the change of direction is greater than 90°.) In addition, the branched calculation was useful in an unintended way: 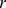 is a behaviorally relevant measure. Reversals are more common during dwelling than during roaming.

#### Calculation

r (reversal), s (speed), and a = (a_t_, a_r_) (acceleration) were calculated as follows. From three consecutive center of mass positions 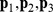 we calculate velocities 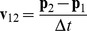, 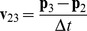. If 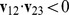, the motion is a reversal, 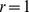, and
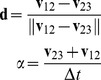
(1)


If 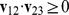, the motion is a non reversal, 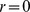, and
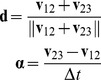
(2)


The unit vector **d** is an estimate of the direction along which the worm is moving. **α** is the laboratory-frame acceleration. To get acceleration along the direction of motion, we rotate **α** through the angle that brings **d** to the *x* axis.
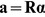
(3)where



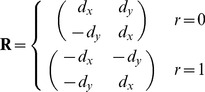
(4)(The negation of the first row of **R** in case of reversal preserves the continuity of 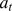 across the switch without changing its magnitude).

 The components of **a** have error on the order of 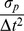 where 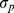 is the position measurement error (typically about 1 µm), even if the worm moves very little between time 1 and time 2, between time 2 and time 3, or in both intervals.

In the case of a reversal, **α** and therefore **a** do not correspond to conventional acceleration, since **α** is based on the sum of the two velocities rather than their difference. For instance, a worm that exactly reverses its movement and ends up at time 3 where it began at time 1 is measured as having **0** acceleration. 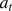 thus corresponds more closely to the common meaning of acceleration, change in speed, rather than the meaning in classical mechanics, change in velocity.

Finally, speed is
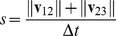
(5)


Together, *r*, *s*, and **a** contain all the translation and rotation-invariant information in 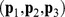
–from *r*, *s*, and **a** three positions can be calculated that differ from 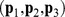 only by translation and rotation.

### Deskewing

The distribution of speed is strongly skewed to the right (Figure S7A). Right skew commonly arises when quantities are better compared by their ratios than their differences, e.g., if one considers speeds of 5 µm s^−1^ and 20 µm s^−1^ to be equally different from 10 µm s^−1^. It is corrected by taking logs. Figure S7B shows the distribution of 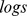. The right skew has been corrected, but there is now a long tail to the left. In the low-speed region, speeds are better compared by differences than ratios: 0.1 µm s^−1^ is very little different from 0.2 µm s^−1^–given the typical noise of position measurement in our system, these are nearly equivalent (assuming a time interval of 1 s). The inverse hyperbolic sine transformation,
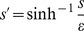
(6)solves this problem: it is linear for 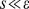 and logarithmic for 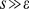 [24].

To allow comparisons between present and future datasets, we did not want 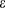 to be a function of data on hand. Instead we chose one value of 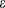 to use for all analyses. Since the switch from logarithmic to linear variation in our data occurs because of measurement error at the low end, we chose 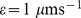, the approximate speed measurement error. Figure S7C shows the results of this transformation. The distribution is not symmetrical, nor is it expected to be, since the distribution of behavior is not, but both long tails have been eliminated.

Magnitude of acceleration 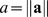 and radial acceleration 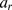 were similarly deskewed using
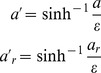
(7)with 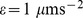.

The results are not sensitive to the precise form of the deskewing transformation. We have also used 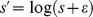, which has the same characteristic of being linear for 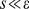 and logarithmic for 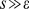. We have also tried different values of 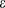; any values of the order of 1 µm s^−1^ (for speed) and 1 µm s^−2^ (for acceleration) gave similar results. For instance, we re-ran the entire analysis leading to Figure 3 with 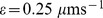 and 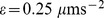, or with 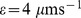 and 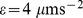. The results were subtly different in predictable ways (smaller values stretch out the low-speed region a little, larger compress it), but none of our conclusions would be altered with this change.

### State Parameters Describing Motion

Based on previous publications [6], [7], [11], we expected that different states would be distinguished by speed and reversal frequency. Previous descriptions of roaming as “sustained” and “straight” [7] suggested (misleadingly, as it turned out) that roaming would be characterized by a low magnitude of acceleration and low radial acceleration. Reversal frequency is characterized by 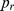, the probability of reversal. Speed and the magnitude of acceleration are characterized by mean speed 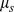, mean acceleration 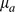, variance of speed 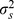, and variance of acceleration 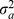. Because we lack information about the dorsal/ventral orientations of the worms and expect them to be random, the mean of radial acceleration was assumed to be zero, so 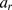 was characterized by its variance 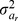 alone. (The zero mean assumption was later tested and found to be correct.) Finally, some preliminary explorations suggested that acceleration and speed might be correlated during dwelling, so we added their covariance 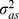 as a seventh parameter. All parameters except 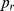 are based on deskewed characteristics.

Using these parameters, we used unbiased closed-loop fitting to identify states as described below. We then calculated 20 parameters for each state: the means, variances, and covariances of *r*, 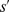, 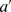, 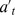, and 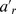. (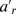 was not assumed to have mean 0 for this analysis, but in fact the measured mean was very close to zero.) We developed a principal component analysis (PCA) browser tool that allowed us to interactively select any subset of these 20 state parameters, carry out PCA on the subset and plot any subset of the experiments in two dimensions with any two of the components chosen as axes, and arrows showing the direction and rate of increase of any subset of the parameters under consideration. Using this tool we systematically surveyed all 20 parameters for those that helped to separate states from tracks recorded under different experimental conditions.

This survey showed that 12 of the 20 parameters were not useful in discriminating states. Including any of these 12 only increased noise. The eight parameters that did help were the original seven plus one more, 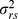, the covariance of reversal and speed. Although this parameter helped when some of the original seven were left out, it didn’t improve discrimination when added to them, i.e., states were not noticeably better separated using eight parameters than seven. We therefore settled on the original seven parameters for further analysis.

### Emission Probabilities

To estimate whether a worm is in a state characterized by the parameters above, we need first to estimate the probability that a worm in that state would exhibit the behavior actually observed (the emission probability). The relationship between *r* and 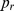 is simple: the probability of 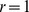 is 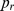; the probability of 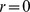 is 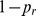. The continuous characteristics *s*, *a*, and 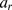 are more complicated. In general, the probability of speed 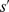 should be high for values close to the mean 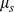 and low for values whose distance from the mean is large compared to the standard deviation 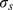. The (unnormalized) probability density of a particular 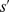 is thus.
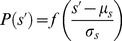
(8)where 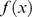 is some positive even function with a maximum at 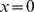 and a value that decreases monotonically as 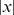 increases. An obvious possibility (and our first choice) for *f* was a Gaussian 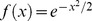; with this choice 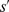 is normally distributed with mean 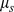 and variance 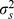. But our attempts at HMM analysis with Gaussian emission distributions failed: no states could be detected that lasted more than a few seconds.

The normal distribution has a perverse property. Consider two states, state D with mean speed 1 and standard deviation 1, and state R with mean 2 and standard deviation 1. If you observe speed 2, you will naturally conclude that the evidence favors state R over D. The extent to which this is true is 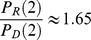. This probability ratio increases as 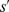 increases:

**Table:** Table 1.

				
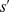 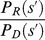	21.65	533.1	104915	20251658240

If speed is normally distributed, a value of 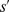 8 standard deviations from the mean is almost 5000 times more probable than one 9 standard deviations from the mean. In the absence of evidence to the contrary, a speed of 10, all by itself, would be sufficient to make you very nearly certain that the worm was in state R rather than D. This, however, is an absurd conclusion, because if speed truly is normally distributed with mean 2 and standard deviation 1, one should not see a speed of 10: the probability is 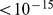. Speeds 8 standard deviations from the mean are not evidence that the worm is in state R–they are evidence that the normal distribution doesn’t correctly describe behavior. Rather, the behavior must be described by a distribution that has much higher probabilities of extreme events.

Such distributions are called fat-tailed distributions. The Student’s *t* distribution is a commonly used symmetric fat-tailed distribution [25]. It corresponds to 
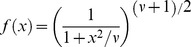
. The parameter ν determines how fat the tails are: smaller ν implies higher probabilities of extreme events. For instance, with 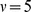 the probability of a speed 8 standard deviations above the mean is about one in 4,000. Such events, while rare, should be seen many times in hundreds of hours of recording. The relative probabilities of R and D with this distribution are:

**Table:** Table 2.

				
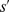 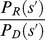	21.73	53.38	101.94	201.38

With this distribution the probability ratio never rises above 3.79, and it actually decreases for extreme speeds. An intuitive interpretation is that a speed of 20 is a poor fit to both states and therefore does not bias one strongly towards one or the other.

The generalization of equation (8) to multiple dimensions is
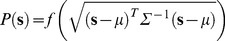
(9)where **s** is the vector of observations, **μ** its mean, and **Σ** its covariance matrix. We chose to use a Student’s *t* distribution with 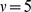, the smallest integer for which the distribution has defined mean and variance in three dimensions. After scaling and normalization, the probability density for 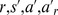 was



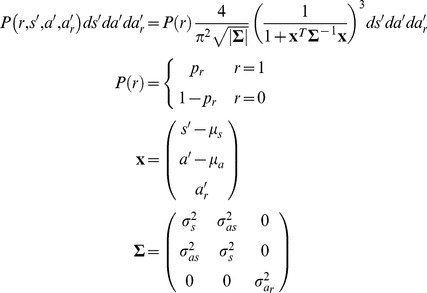
(10)This distribution is at best an approximation to the true distribution. For instance, it has a finite probability of negative speed, even though speed by definition is never negative.

### Transition Probabilities

HMM fitting is the process of looking for an explanation of the worm’s behavior in terms of changes in its behavioral state. Two things determine the posterior probability that the worm is in state *i* at time *t*. The first is the probability that a worm in state *i* would display the behavior actually observed at time *t*, i.e., how well is behavior explained by state? This is the emission probability just described. The second is the probability that it could have entered or remained in state *i* at time *t*, given the state probabilities at the previous and subsequent time points. If the time between subsequent points is short, the worm should usually stay in the same state. Thus, explanations in which state changes are rare are preferred (Figure 2A,B). It is this second criterion that allows HMM fitting to make use of information about how behavior is arranged in time.

The penalty for switching between states is governed by a lifetime τ. The transition probability matrix **T** from time *t* to time 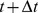 is
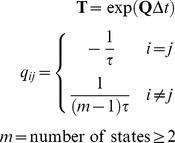
(11)


τ is a tuning parameter that can be used to trade stability for rapid response. Formally it is the mean lifetime of a state in the underlying Markov chain. However, in HMM fitting it is better thought of as the weight of behavioral evidence required to recognize a state switch. For most of our analysis we used 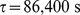 (one day). With this value and 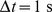 behavioral evidence for a switch from state 1 to state 2 must be sufficient to overcome an 86,400 fold penalty against switching. Such evidence could be supplied by three points in which the behavior has a 50-fold higher probability of occurring in state 2 than in state 1 (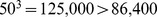). We chose 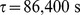 so that a single outlying point would not be sufficient to force a state switch. However, rapid changes in behavior might be missed with this large a value of τ. For the starvation recovery experiment, in which we were looking for rapid changes of behavior, we used 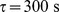 (5 min).

τ does not constrain the actual lifetimes observed. If 50-fold differences in emission probabilities are common (and they were), a state switch can be recognized in as little as 3 s. In fact, with 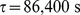 the typical lifetimes observed were between 1 and 2 min. Furthermore, results were fairly insensitive to this setting. As a check, we re-ran the entire analysis leading to Figure 3 with 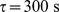. The results are only subtly different, and none of our conclusions are changed. Even with 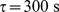, typical observed lifetimes were about 30 s.

### Parameter Estimation

Given the seven parameters describing each state, a track can be fit to an HMM by the forward-backward algorithm [26, Section 16.3]. From the fit one obtains the likelihood that a worm whose behavior was governed by the model would behave as this worm actually did, as well as a set of state probabilities for each point.

In general, the behavior of the worm in state *i* in a particular track will not precisely match that described by the input parameters. For example, suppose that a track is fit to a model with two states, state R with mean speed 2 and state D with mean speed 1. However, this worm is actually switching between a state with mean speed 3 and another with mean speed 0.5. Despite the mismatch between the model and actual behavior, the fit will detect the alternation between states, since state R will fit better to the speed 3 state than state D does, and state D will fit better to the speed 0.5 state than state R does. In this case, by looking at the actual behavior during the times when state R was determined to be more probable, the true characteristics of the high-speed state can be estimated.

Actual behavior was estimated from the fit as the average of functions of motion characteristics, weighted by the state probabilities:
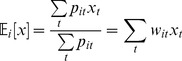
(12)where



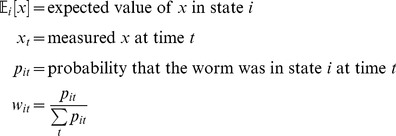
(13)
Equation (12) is a straightforward extension of the Baum-Welch method for estimating the symbol probability matrix for an HMM with discrete emissions [26]. Intuitively, it says that those time points at which the worm has a high probability of being in state *i* are weighted heavily in estimating the characteristics of state *i*.

We estimated parameters 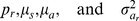 in this way, using
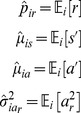
(14)


The variances and covariance 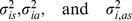 were estimated using
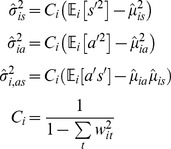
(15)



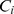
 corrects for the bias introduced by using estimated means instead of the (unknown) true means in estimating variances. This correction is unnecessary for 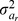 because the mean of 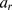 is assumed zero.

### Unbiased Closed-loop Fits

Given guesses of the behavioral states, an HMM fit can be performed on a track and new estimates calculated as described above (an *open-loop fit*, Figure S3A). Instead of stopping there, however, one can feed these new estimates into a second HMM fit of the same track to obtain a third set of estimates, and so on. Under favorable conditions the estimates will eventually stop changing. We call this a *closed-loop fit* (Figure S3B). Some adjustments were necessary to achieve convergence in closed-loop fits. First, we do not use re-estimated transition probabilities, but constrain them to the form described above. Second, we do not allow the variance parameters to vary independently for the separate states, but instead calculate a single value for each of these as a weighted average of the estimates for the separate states. Because of the form of the distribution we assumed, this ensured that the ratios of emission probabilities were bounded. After convergence, we run the states through a single round of open-loop fit with these constraints relaxed so as to estimate independent variance parameters for each state. We also did a single step of the Baum-Welch algorithm [26] for re-estimating the transition matrix **T**.

The closed-loop fit still requires initial guesses of state parameters to get started, and it is conceivable that these initial guesses might influence the states eventually discovered. In an *unbiased closed-loop fit*, initial guesses derived entirely from the data (Figure S3C). We began by fitting the data to a one-state model. In this case no initial guess is necessary, since the worm must be in the single state with probability 1 during the entire track. We then split the state into two, one identical to that derived from the one-state fit, and a second with slightly greater mean speed, and used these as the initial estimates for a two-state closed-loop fit. Although the fit began with two almost identical states, the slightly higher-speed state has higher probability during portions of the track when the worm is moving faster and the lower-speed state when the worm is moving slower. If there are coherent behavioral variations, the low and high-speed states will therefore take on different characteristics on parameter re-estimation, and during subsequent iterations they converge on different parts of the track. If the two-state fit converged, its higher-speed state was split in the same way to produce initial guesses for a three-state fit. Goodness of fit, measured by likelihood, tended to increase with more states: log-likelihood per point increased by 0.34±0.20 (mean ± standard deviation; range 0.013–1.14, 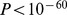, signed rank test) in going from one to two states, and 0.069±0.065 (range −0.016–0.35, 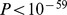). (In 18/363 cases likelihood decreased slightly in going from two to three states. It is not surprising that likelihood decreased slightly in some cases, since the unbiased parameter estimates (14), (15), are not maximum likelihood estimates. The equal variance constraint can also prevent achieving maximum likelihood.) We didn’t try to continue past three states, since in most three-state fits there was at least one in which the worm spent little time. The fit with the highest excess entropy was used for further analysis.

Although we refer to these fits an “unbiased”, we recognize that this description is relative. Any method of recognizing behavioral patterns will of course be biased by the data collected. More subtly, to use the method it is necessary to reduce possible patterns of behavior to numerical descriptions, as described above. There is no fixed recipe for developing such a description scheme, and it determines what sort of patterns can be recognized.

### Standard State Descriptions and Fits

While unbiased closed-loop fits capture a lot of information about an individual worm’s movement, they are difficult to compare to published results. We therefore developed standard roaming, dwelling, and quiescence state descriptions that could be used for fitting all tracks. While these standard state fits probably do not classify behavior as accurately as unbiased fits, they have the advantage of describing behavior in familiar terms.

Based on past results, we identified *pure plays*–conditions under which a worm spends most of its time in one of the three states. These conditions were:

Roaming: well-fed wild-type worms on poor food, well-fed *egl-4* loss-of-function mutant worms on poor and medium-quality food. Poor food is *E coli* HB101 grown on aztreonam [7]. Medium-quality is a mixture of aztreonam-treated and untreated. Poor food suppresses dwelling and quiescence, and *egl-4* is necessary for both [1], [6], [11].

Dwelling: well-fed *ttx-3*, *tax-4*, and *daf-7* loss-of-function mutant worms on good food (*E coli* HB101); *daf-7* loss-of-function mutant worms fasted for 12 h, then refed for 3 h on good food. Under our recording conditions well-fed worms show little quiescence on good food. *ttx-3* and *tax-4* are necessary for normal levels of roaming [1], [7]. *daf-7* worms have been reported to be defective in both roaming [7] and quiescence [11].

Quiescence: *egl-4* gain-of-function mutant worms fasted for 12 h, then refed for 3 h on good food [10], [11].

Unfortunately, none of these is a perfect pure play. We therefore chose the most probable state from the unbiased closed-loop fit of each track as the basis for pure-play state descriptions. The seven parameter values were calculated as described under Parameter estimation, except that in
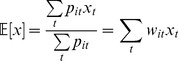
(16)

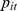
 now refers to all time points in all the tracks being pooled to produce a state description, and i is the most probable state in each track. Parameters 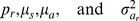 of the pure play states are thus the weighted average of the means of the corresponding parameters of the most probable states in the constituent tracks. The variances 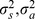 and the covariance 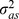 are larger, however, since they are based on the common means over all tracks rather than the within-track means.

Two kinds of effects can be detected in standard state fits. First, a treatment or genotype may affect the rate at which a worm switches between roaming, dwelling, and quiescence. Second, the treatment may affect the way a worm behaves when in a particular state. For instance, it has been suggested, and we confirmed, that roaming worms move faster on low-quality food [7]–this effect is in addition to the increase in the frequency of roaming. Interpretation of these fits is complicated by the fact that one effect can masquerade as the other. For instance, if in some genotypes dwelling worms behave in ways that are closer to quiescent worms, this may appear as an increase in the frequency of quiescence.

### Statistically Typical Tracks

The short illustrative statistically typical segments in Figure 1 in were chosen as follows. First, the most probable states from unbiased closed-loop fits on which the corresponding standard state description was based (see above) were averaged to get the target state. Next, the state descriptions were standardized to have standard deviation 1, and that state and track that yielded a standardized description closest to the mean were chosen. Finally, the central 90 s from the longest segment within this track in which the probability of being in this state remained continuously at 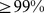 was chosen.

### Excess Entropy

Excess entropy in bits per point for an *m*-state fit to a track was calculated as
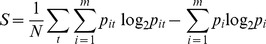
(17)


*N* is the number of time points, and 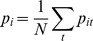 is the probability of state *i* in the track as a whole. Since 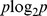 is convex, 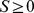 by Jensen’s inequality. The maximum possible value, 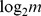, occurs when all states are equally probable in the track as a whole, but at each time point one state has probability 1. This maximum is 1 bit for a two-state fit and 1.58 bits for a three-state fit.

An analogy will help to understand the meaning of excess entropy. Suppose there are three weather forecasters. Forecaster 1 says the probability of rain is 1/7 every day this week. Forecaster 2 says the probability of rain is 1/12 Sunday through Friday and 1/2 Saturday. Forecaster 3 says the probability of rain is 0 Sunday through Friday and 1 Saturday. Suppose too that over the long term all three forecasters are statistically accurate, e.g., it rains on one out of twelve of those days on which Forecaster 2 says there is a 1/12 probability of rain. Despite the equal accuracy of all three forecasts, Forecaster 2′s prediction is more informative than that of Forecaster 1, and Forecaster 3′s prediction is more informative still. The excess entropy of Forecaster 2′s prediction is 0.094 bits/day: on the average, she provides 0.094 bits more information each day than Forecaster 1. The excess entropy of Forecaster 3′s prediction is 0.59 bits/day. Excess entropy is a measure of precision, not accuracy. The excess entropy of Forecaster 3′s prediction is 0.59 bits/day even if it rains on Friday and not Saturday.

### Principal Components, Cluster Analysis, and Triangularity Test

We used principal component analysis (PCA) to reduce the dimension of states discovered by unbiased closed-loop fits as an aid in visualization. These fits often gave rise to outlying states of low probability. (These are visible in Figure 3GH as small dots surrounding the main body of states; in 3 dimensions they are even more evident.) To reduce the influence of these low-probability states we weighted the PCA by state probability. Let 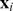 be the parameter 7-vector describing state *i* and 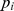 the probability of that state in the track in which it occurred as a whole. We defined expectation over this set by
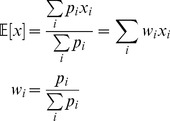
(18)
*i* runs over all states in all tracks. Means, variances, covariances, and correlations were then defined in the usual way using this expectation, and principal components were calculated by diagonalizing the correlation matrix so defined.

For hierarchical clustering, we first standardized all 1083 states so that each parameter had mean zero and standard deviation 1 by the weighted measure (18). We then eliminated all states with probability less than 10%, leaving 832, which were clustered using the Hierarchical Clustering package in Mathematica (Wolfram Research). Cluster analysis was done using distance measure Euclidean Distance or Euclidean Distance Squared and all available linkage methods. Results varied greatly depending on these settings. We got the best results with Euclidean Distance Squared and linkage method Median; these are shown in Figure S4. Results were also sensitive to the inclusion or exclusion of particular datasets–often the inclusion of a few new recordings caused a near complete rearrangement of the clusters.

Triangularity was tested as described by Shoval et al. [13], with small modifications. The test is based on a statistic *t*, the ratio of the area of the convex hull of the points in two dimensions (i.e., the first two principal components) to that of the smallest triangle that contains them. For points arranged in a triangle, this ratio is 1; for a more compact arrangement, it is smaller than 1. We tested significance by randomly permuting one component, so that the permuted data contained the same *x*’s and *y*’s, but associated with each other randomly. (This is slightly different from Shoval et al. [13], who derived new points by independently resampling the *x* and *y* distributions, but the central idea of scrambling the association between *x* and *y* is the same.) We sampled either 10^4^ or 10^5^ permutations to determine the distribution of *t*. We first tested the full set of 1083 states and found that they were significantly triangular with 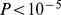. The test is sensitive to outliers, so we repeated it on the set of 832 states with probability at least 0.1 used for cluster analysis, which was also significant triangular at 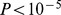.

### Fit and State Dissimilarity

We developed measures of the difference in behavior between two worms based on HMM analysis. The fundamental logic is simple: if worm A and worm B behave similarly, the model that best describes worm B’s behavior should also do well at describing worm A. We use log likelihood per point as a measure of goodness of fit. Define
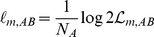
(19)

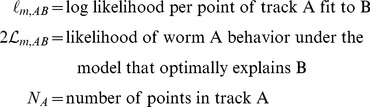
(20)


Optimal models come from unbiased closed-loop fits.

Model dissimilarity between A and B is then
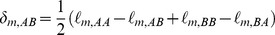
(21)


Model dissimilarity thus defined doesn’t properly account for differences in state probabilities. For instance, suppose that worm B is optimally described by a two-state model in which state 1 has a lifetime 1.5 times that of state 2, so that the worm is expected to spend 60% of its time in state 1 and 40% in state 2. Worm A, on contrast, spends 100% of its time behaving as expected in worm B’s state 1. In this case, 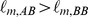; worm A better fits B’s model than B itself does. A simpler example illustrates why this happens. Suppose you have a biased coin that comes up heads 60% of the time. The most likely sequence, if you flip it 100 times, is 100 heads in a row. This is more likely than any single sequence of 60 heads and 40 tails by a factor of 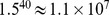. Model likelihood fails to account for the fact that there are many sequences of 60 heads and 40 tails, 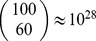 of them, compared to just one sequence of 100 heads.

Let 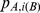 be the probability of worm B’s state *i* in the behavior of worm A. If worm A’s behavior were statistically identical to worm B’s, the likelihood of particular values of 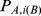 would be described by the multinomial distribution,
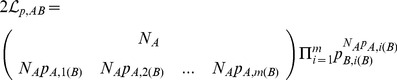
(22)


Defining log likelihood per point analogously to (19), one finds that in the limit as 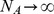,
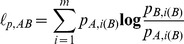
(23)



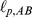
 is 0 when the 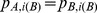 and negative for any other 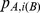. State probability dissimilarity 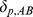 is defined analogously to (21). Fit dissimilarity, our measure the similarity of behavior, is the sum of model dissimilarity and state probability dissimilarity
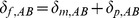
(24)


(24) may overweight state probabilities, since it effectively treats each time point as independent, although in reality behavior at nearby times is correlated. However, it is our impression that the *ad hoc* emission distribution (10) overweights the match of behavior to state parameters. We consider the correct weighting of 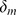 and 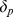 to be an empirical question, rather than a theoretical one. In practice, (24) produces useful results (Figure 5, Figure S5).

We also designed a related measure, state dissimilarity, in an attempt to measure only the difference in state characteristics. State dissimilarity 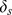 is defined like 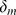, except that in computing 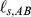 the transition frequencies estimated from model B are not used. Instead, worm A is fit to a model in which the state parameters are those of model B, and the transition matrix is as given by eq (11), using the same value of τ as for the closed-loop fit used to determine model B. No probability dissimilarity is used. The effect is that the only information from B used in the fit of A is the state parameters. Unfortunately, this measure does not perfectly separate state characteristics from state probability. Suppose worms A and B have identical states, but the probability of state 1 is high in worm A and very low in worm B. It may be that worm B does not enter state 1 at all during the course of a recording, so that its characteristics can’t be estimated. In this case worm A will fit poorly to B’s model, 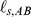 will be small and 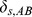 large, even though the two worms have identical states.

### Multidimensional Scaling

We used multidimensional scaling for two purposes: first, at the request of one reviewer, to check the configuration of the state space shown in Figure 3, and second, to visualize fit dissimilarity within and between experiments (Figure S5). For the first purpose, the distance 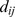 between states *i* and *j* was the Euclidean distance between their parameter vectors, standardized as for hierarchical clustering above. Starting from locations defined by the first components of principal component analysis, we then searched for locations 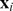 that minimized the potential
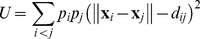
(25)


The sum runs over all pairs of different states. As above, 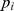 is the probability of state *i* in the track in which it occurred as a whole. The stress for the optimal locations, defined as
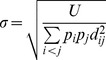
(26)was 0.047 in two dimensions. (Stresses below 0.1 are typically considered acceptable.) The optimal configuration of states (not shown) was only subtly different from that obtained by principal component analysis (Figure 3).

For the second purpose, multidimensional scaling was done on the 363 tracks, rather than the 1083 states. We began with fit dissimilarity, 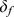 (eq (24)). Fit dissimilarity is not a distance metric, since it doesn’t satisfy the triangle inequality 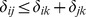. In addition, it can with low probability be negative. (Of the 90,679 dissimilarities calculated for this paper, one was very slightly negative, at −0.0007.) Thus, we first derived a distance metric from fit dissimilarity.

We combined two methods of eliminating triangle violations. The first was simply to add a constant to all dissimilarities. (This also fixes the negative dissimilarity.) Unfortunately, the constant required if this method was used alone would have been 6.28. Since the median within-experiment dissimilarity was 0.28, this would have destroyed close relationships, making every track very distant from every other. The second method was to let 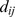 be the weighted graph distance from track *i* to track *j* on a graph with edge weights 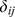. That is, the distance from *i* to *j* is the length of the shortest path from *i* to *j*, whether direct or via other tracks. This method, used alone, had the opposite problem: it made distant things too close. The combined method we finally adopted was to use weighted graph distance with edge weights 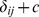. Since our goal was to find 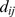 that mimicked the 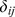 as closely as possible, we chose the constant *c* to maximize the reflective correlation between 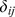 and 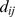.
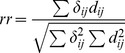
(27)


The optimal value of *c* was 0.071, safely less than 0.28.

The 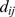 computed, the remaining steps were conventional, using Torgerson’s double-centering method to derive an initial configuration, then optimizing an unweighted spring potential.
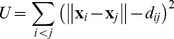
(28)


Optimized stresses were 0.072 in two dimensions and 0.044 in three. The optimal two-dimensional configuration is shown in Figure S5, but the third dimension from the optimal three-dimensional configuration was used to determine the order in which points were plotted, to give a sense of front and back.

### Starvation Recovery Statistical Tests

For the starvation recovery experiment we needed to test whether certain mean dissimilarities (same/different worm, same/different time) depended on either *t* or 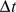. There are several ways to test this–the values reported are those from the most conservative test, which was based on the significance of the slope parameter in a linear regression of mean dissimilarity against time or time interval. Other tests, e.g. rank-based tests such as Spearman’s rank correlation, gave smaller (i.e., more highly significant) *P* values. Although the points involving the 0–15 min cuts diverged significantly from the others and are highlighted in Figure 5, they were included in the test on an equal footing. *P* values were lower if they were excluded.

The *P* values we report for dependence on 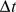 exclude 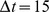
_. Consider, for example,_ the 15–30 min cut and the 30–45 min cut. They are contiguous in time and are extracted from one continuous recording. Unless the worm happened to change state between 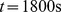 and 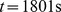, it would have begun the 30–45 min cut in the same state in which it ended the 15–30 min cut. This would lead to a similarity between these segments that might not reflect ongoing changes in the underlying drivers of the worm’s behavior. We thus excluded comparisons between adjacent cuts from statistical tests, so that the tests only included cuts whose end and beginning were separated by at least 15 min. The mean state lifetime in these experiments was 35 s; the probability that a state with lifetime 35 s persists for 15 min is 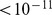. *P* values were lower if 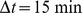 points were included.

### Software

MATLAB and Mathematica scripts developed for this analysis are available at http://elegans.som.vcu.edu/~leon/HMM.

## Supporting Information

Figure S1 **Roaming, dwelling, and quiescence speed histograms.** All 363 tracks were analyzed by open-loop fits to the standard roaming, dwelling, and quiescent state descriptions defined by standard state analysis, then the time points were selected at which one state was assigned with at least 99% probability. At each such point we determined center of mass speed and change in direction. This histogram plots speed alone; Figure S6 shows both speed and direction change. Blue is quiescence, green dwelling, and red roaming. To allow all three distributions to be clearly seen, the plot was cut off at 0.2. The probability of *s* <5 µm s^−1^ for quiescence is 0.76.
(TIF)

Figure S2 **Speed and direction change for roaming, dwelling, and quiescent worms.** For each point classified as described in the legend to Figure S1, we determined speed and absolute change in direction of the center of mass. In all states the direction change is concentrated near 0° and near 180°, with a wider spread at low speeds as expected from the difficulty of accurately measuring directions when movements are small. Our motion analysis classifies as reversals those points with a direction change greater than 90°.
(TIF)

Figure S3 **Open-loop, closed-loop, and unbiased closed-loop fits.** A. In an open-loop fit, a behavioral record is fit to a hidden Markov model based on states with predefined characteristics. The results are then used to re-estimate the characteristics of the behavior actually observed. However, because the state classification is based on the predefined states, the re-estimated state characteristics will tend to resemble those of the predefined input states. B. In a closed-loop fit, the fit is repeated with re-estimated state characteristics. This process is repeated until the estimates stop changing. C. In an unbiased close-loop fit, initial estimates are derived from the behavioral record itself.
(TIF)

Figure S4
**Hierarchical cluster analysis of states.** A. Hierarchical clustering of state descriptions resulting from unbiased closed-loop fits. 832 of the 1083 states plotted in Figure 3G, those with probability 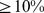, were clustered. The seven values constituting each description are plotted in the heat map below the dendrogram, and the top three clusters are highlighted in blue, green, and red. B. Identification of clustered states. States, plotted as in Figure 3, are identified by red, green, and blue dots according to which cluster they belong to.(TIF)

Figure S5 **Under similar conditions, worms behave similarly.** Each of the 363 dots in each of the 49 panels represents a single worm. In each panel tracks from one experiment are highlighted in color. The text above each panel corresponds to its ID in Table S1. The color assigned to each experiment is the same as in Figure 3. The dots are arranged in two dimensions so that those representing similar behavior are closer to each other than those representing dissimilar behavior (see Multidimensional scaling in Methods). A third dimension is hinted at by the partial obscuring of some dots by others in front of them.
(TIF)

Figure S6
**State changes during recovery from starvation.** Each panel shows the states of 14 worms recovering from starvation during one 15 min interval, plotted as in Figure 3. The gray background shows all states from the 49 experiments in Table S1–it is slightly different from Figure 3 because these experiments were analyzed with lifetime parameter 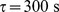. Each worm is assigned a different color.(TIF)

Figure S7
**Deskewing speed.** A. A histogram of speeds from all tracks combined. The distribution is strongly skewed to the right. B. Histogram of the logarithm of speed. This distribution shows a tail to the left. C. Histogram of speed deskewed using 
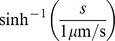
. Both tails have been eliminated.(TIF)

Table S1 **Experiments analyzed.**
(DOC)

Dataset S1 **State probabilities and transition rates.**
(XLSX)

Movie S1 **Well-fed wild type (30 min).** The following 4 video files are provided as examples of behavioral states. Each video shows the movements of the center of mass of a single worm over 30 or 60 min at 15 times actual speed. Behavioral state (based on a fit to standard states) is coded by color. 100% probability of roaming, dwelling, and quiescence are shown as pure red, green, and blue, respectively, and times at which more than one state has finite probability are shown as mixtures of these three colors. A black ring indicates a reversal. Empty circles are times for which we do not have data; state and position are interpolated at these times. Movie S1 is the track analyzed in Figure 2C.
(MOV)

Movie S2 **Wild type, fasted and refed (60 min).**
(MOV)

Movie S3 **Wild-type on medium-quality food (30 min).**
(MOV)

Movie S4 **egl-4(gf) fasted and refed (30 min).**
(MOV)
