# Supplementary figures and images for: The Geometry of Locomotive Behavioral States in C. elegans
Source: PLoS One. 2013 Mar 28;8(3):e59865. doi: 10.1371/journal.pone.0059865 (PMC3610905; doi:10.1371/journal.pone.0059865)

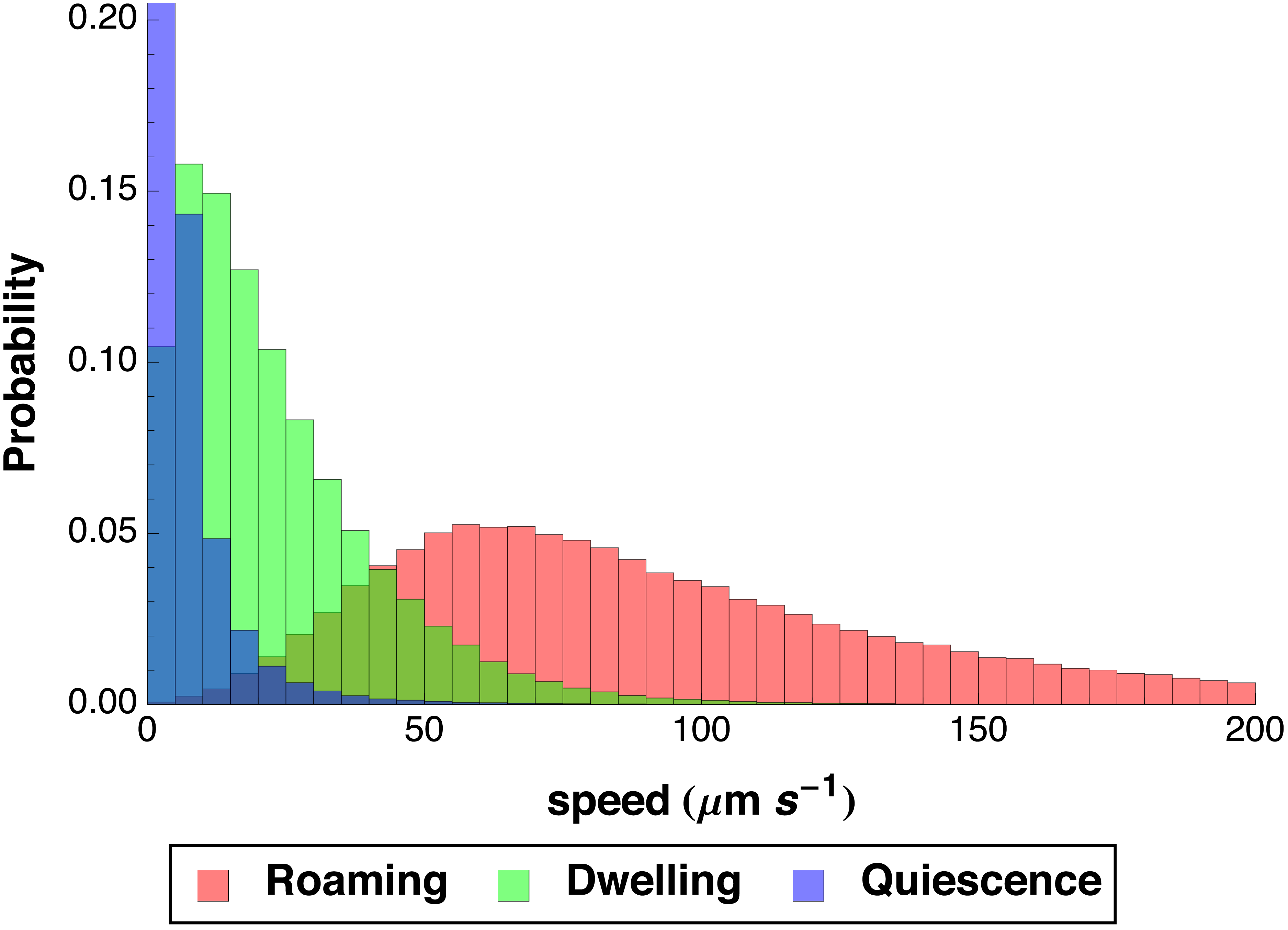

Supplement: Figure S1 — Roaming, dwelling, and quiescence speed histograms. All 363 tracks were analyzed by open-loop fits to the standard roaming, dwelling, and quiescent state descriptions defined by standard state analysis, then the time points were selected at which one state was assigned with at least 99% probability. At each such point we determined center of mass speed and change in direction. This histogram plots speed alone; Figure S6 shows both speed and direction change. Blue is quiescence, green dwelling, and red roaming. To allow all three distributions to be clearly seen, the plot was cut off at 0.2. The probability of s <5 µm s−1 for quiescence is 0.76. (TIF) [file pone.0059865.s001.tif]

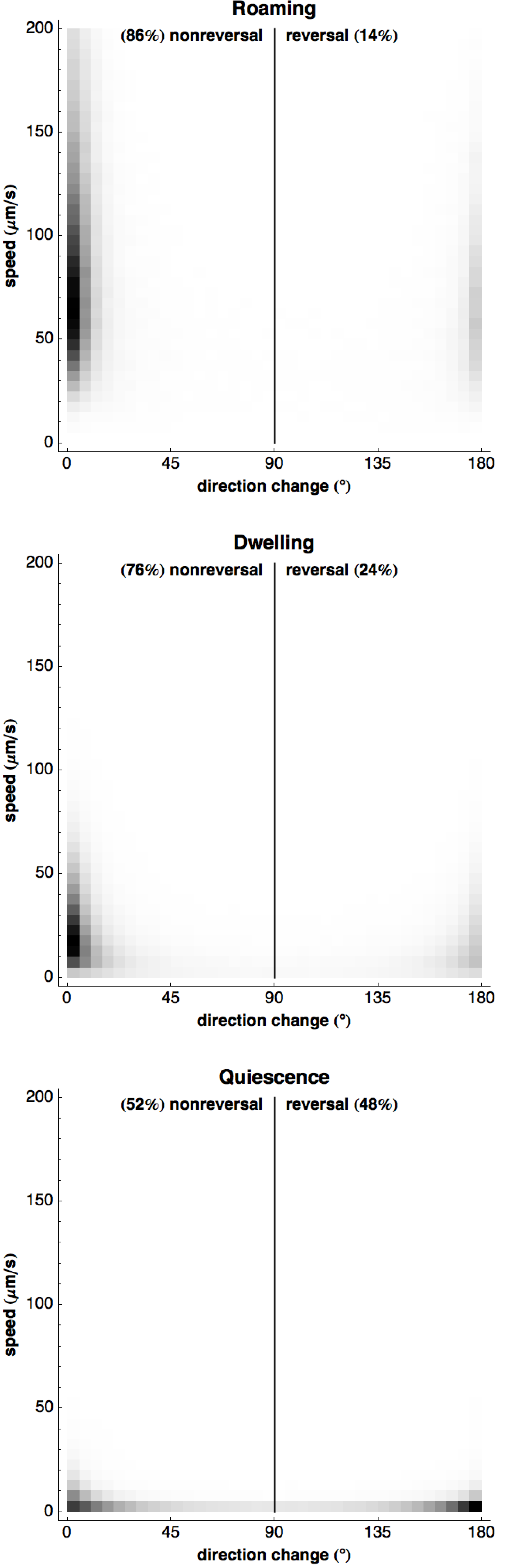

Supplement: Figure S2 — Speed and direction change for roaming, dwelling, and quiescent worms. For each point classified as described in the legend to Figure S1, we determined speed and absolute change in direction of the center of mass. In all states the direction change is concentrated near 0° and near 180°, with a wider spread at low speeds as expected from the difficulty of accurately measuring directions when movements are small. Our motion analysis classifies as reversals those points with a direction change greater than 90°. (TIF) [file pone.0059865.s002.tif]

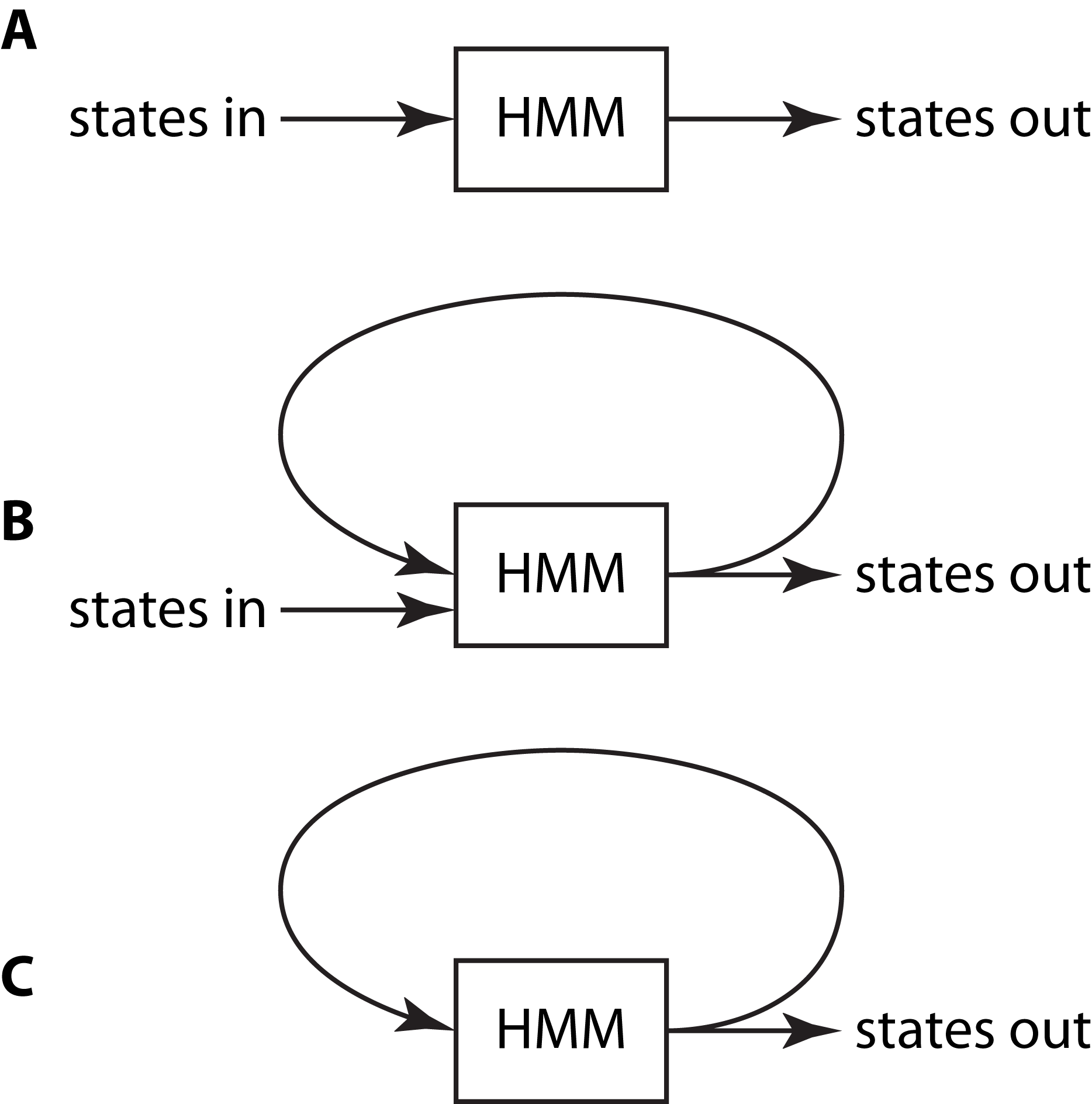

Supplement: Figure S3 — Open-loop, closed-loop, and unbiased closed-loop fits. A. In an open-loop fit, a behavioral record is fit to a hidden Markov model based on states with predefined characteristics. The results are then used to re-estimate the characteristics of the behavior actually observed. However, because the state classification is based on the predefined states, the re-estimated state characteristics will tend to resemble those of the predefined input states. B. In a closed-loop fit, the fit is repeated with re-estimated state characteristics. This process is repeated until the estimates stop changing. C. In an unbiased close-loop fit, initial estimates are derived from the behavioral record itself. (TIF) [file pone.0059865.s003.tif]

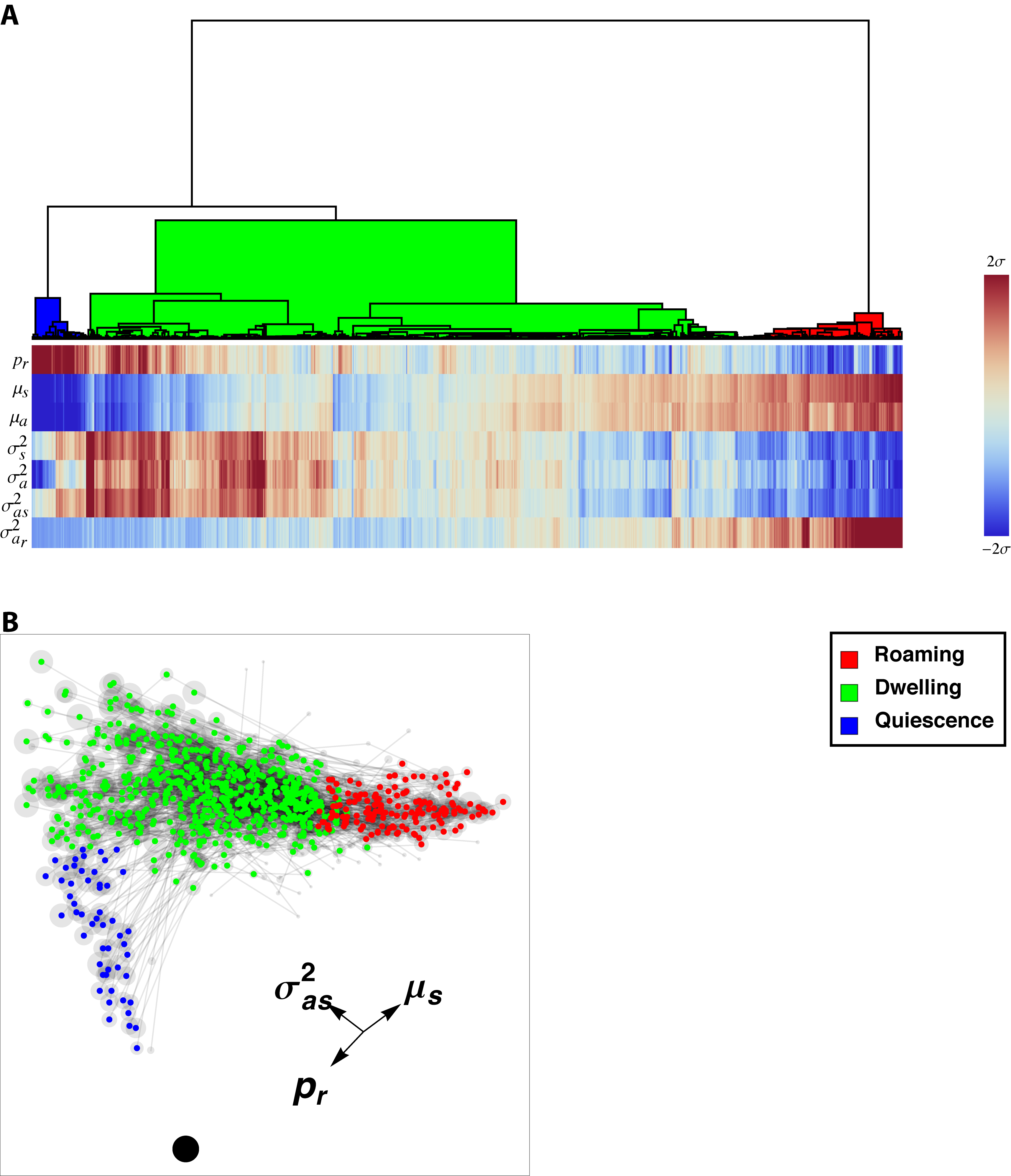

Supplement: Figure S4 — Hierarchical cluster analysis of states. A. Hierarchical clustering of state descriptions resulting from unbiased closed-loop fits. 832 of the 1083 states plotted in Figure 3G, those with probability , were clustered. The seven values constituting each description are plotted in the heat map below the dendrogram, and the top three clusters are highlighted in blue, green, and red. B. Identification of clustered states. States, plotted as in Figure 3, are identified by red, green, and blue dots according to which cluster they belong to. (TIF) [file pone.0059865.s004.tif]

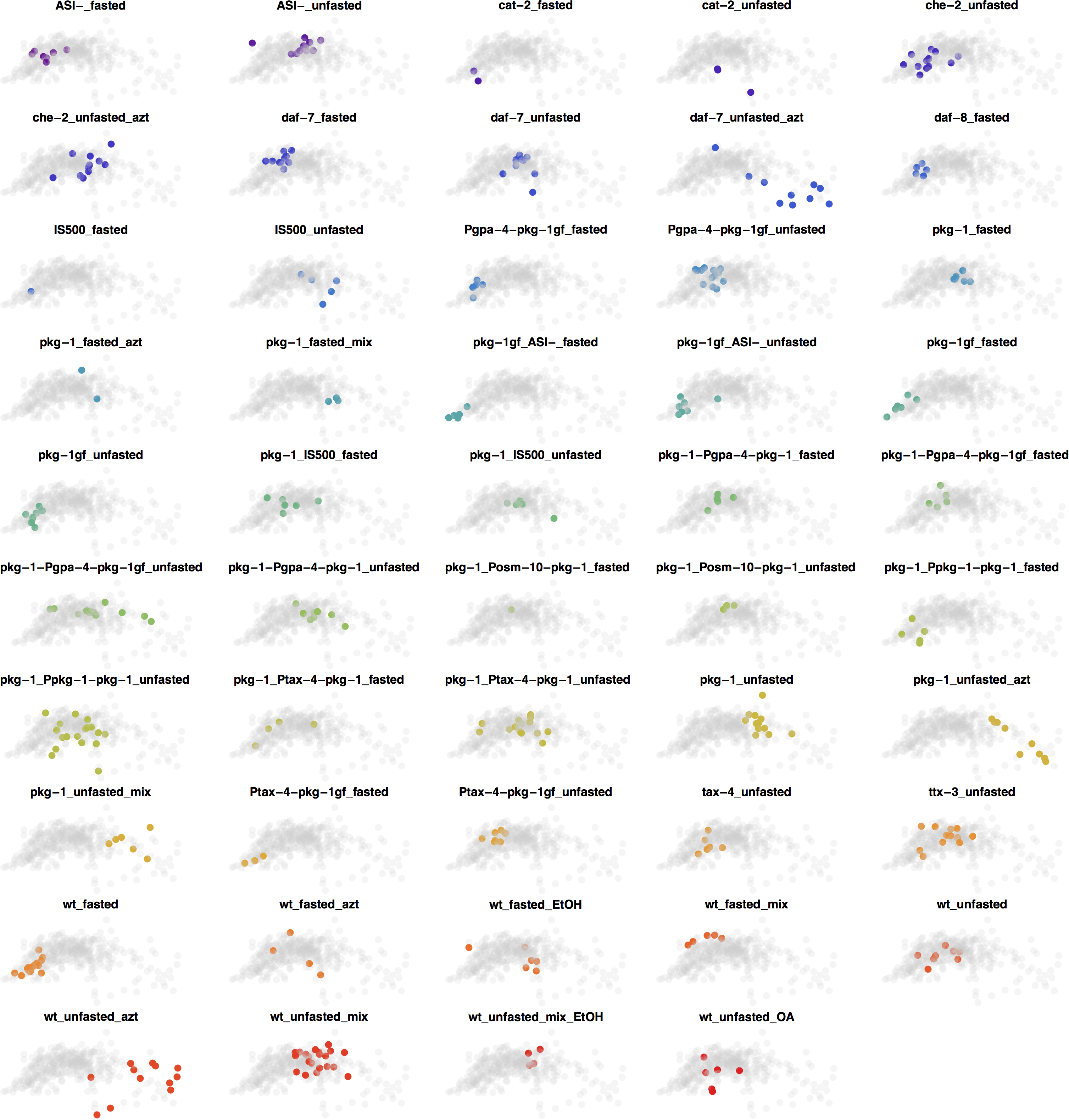

Supplement: Figure S5 — Under similar conditions, worms behave similarly. Each of the 363 dots in each of the 49 panels represents a single worm. In each panel tracks from one experiment are highlighted in color. The text above each panel corresponds to its ID in Table S1. The color assigned to each experiment is the same as in Figure 3. The dots are arranged in two dimensions so that those representing similar behavior are closer to each other than those representing dissimilar behavior (see Multidimensional scaling in Methods). A third dimension is hinted at by the partial obscuring of some dots by others in front of them. (TIF) [file pone.0059865.s005.tif]

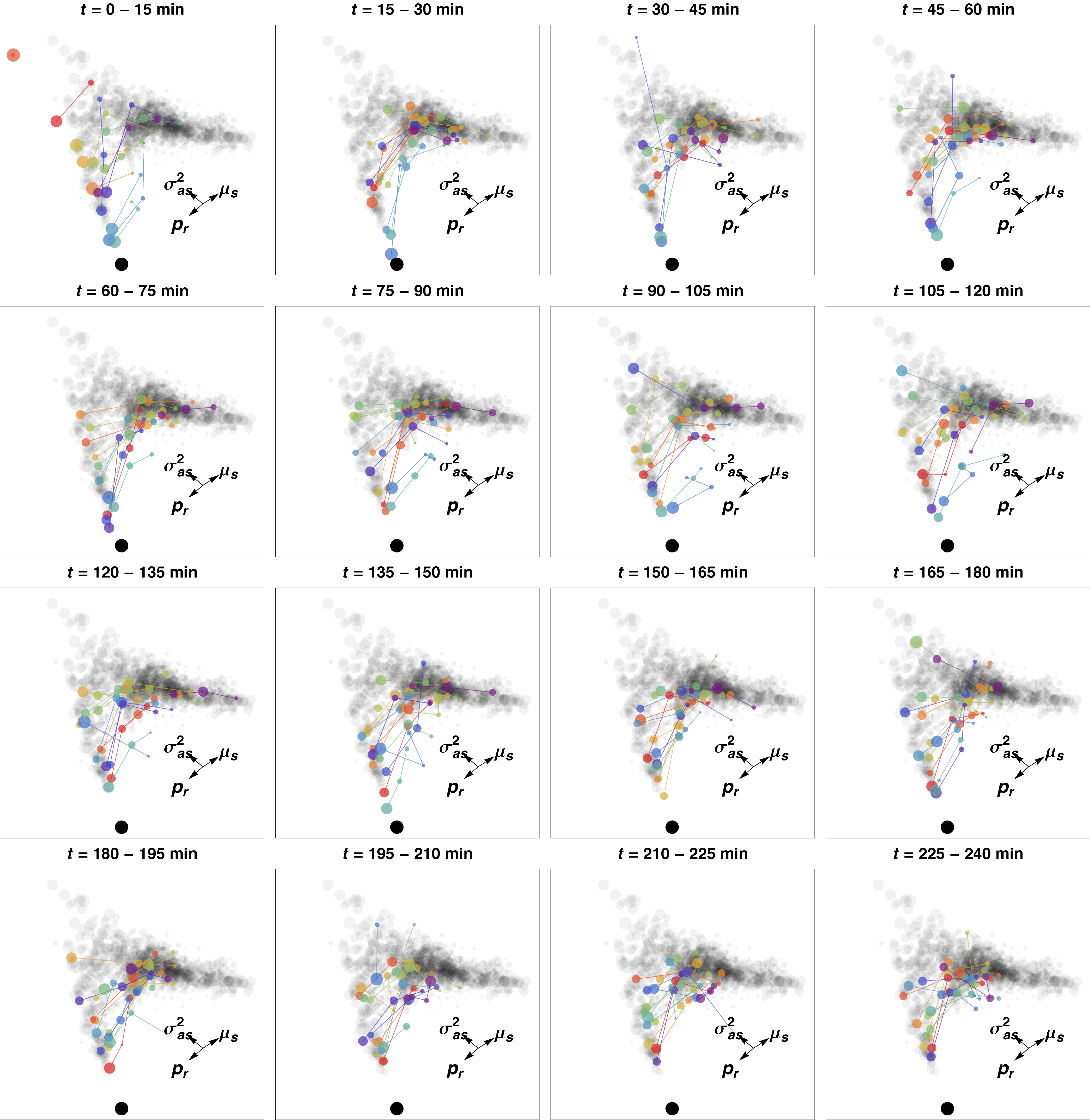

Supplement: Figure S6 — State changes during recovery from starvation. Each panel shows the states of 14 worms recovering from starvation during one 15 min interval, plotted as in Figure 3. The gray background shows all states from the 49 experiments in Table S1–it is slightly different from Figure 3 because these experiments were analyzed with lifetime parameter . Each worm is assigned a different color. (TIF) [file pone.0059865.s006.tif]

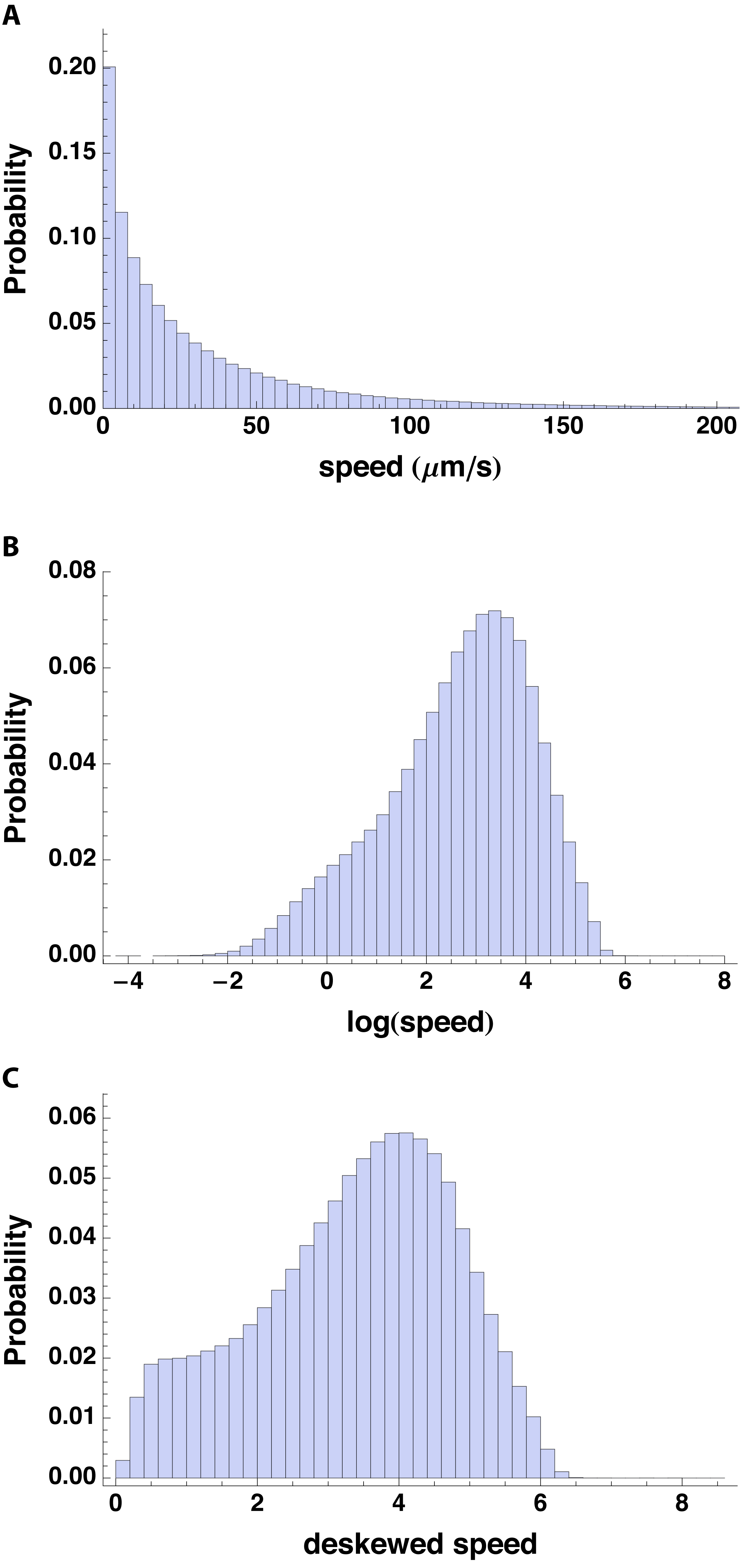

Supplement: Figure S7 — Deskewing speed. A. A histogram of speeds from all tracks combined. The distribution is strongly skewed to the right. B. Histogram of the logarithm of speed. This distribution shows a tail to the left. C. Histogram of speed deskewed using . Both tails have been eliminated. (TIF) [file pone.0059865.s007.tif]
