# Supplementary material for: The Geometry of Locomotive Behavioral States in C. elegans
Source: PLoS One. 2013 Mar 28;8(3):e59865. doi: 10.1371/journal.pone.0059865 (PMC3610905; doi:10.1371/journal.pone.0059865)
Supplement: Table S1 — Experiments analyzed. (DOC) [file pone.0059865.s008.doc]

# Supporting Table

Table S1: Experiments analyzed

| **ID** | **genotype** | **feeding history** | **food^1^** | **N** |
| --- | --- | --- | --- | --- |
| ASI-_fasted | *Is[gpa-4p::recCaspase gcy-27p::recCaspase gcy-27p::gfp]*^2^ | fasted/refed | normal | 7 |
| ASI-_unfasted | *Is[gpa-4p::recCaspase gcy-27p::recCaspase gcy-27p::gfp]*^2^ | well-fed | normal | 11 |
| cat-2_fasted | *cat-2(e1112) II* | fasted/refed | normal | 2 |
| cat-2_unfasted | *cat-2(e1112) II* | well-fed | normal | 3 |
| che-2_unfasted | *che-2(e1033) X* | well-fed | normal | 12 |
| che-2_unfasted_azt | *che-2(e1033) X* | well-fed | Azt | 11 |
| daf-7_fasted | *daf-7(e1372) III* | fasted/refed | normal | 10 |
| daf-7_unfasted | *daf-7(e1372) III* | well-fed | normal | 10 |
| daf-7_unfasted_azt | *daf-7(e1372) III* | well-fed | Azt | 10 |
| daf-8_fasted | *daf‑8(e1393) I* | fasted/refed | normal | 5 |
| IS500_fasted | *pyIs500[odr-3p::gfp::egl-4]*^3^ | fasted/refed | normal | 1 |
| IS500_unfasted | *pyIs500[odr-3p::gfp::egl-4]*^3^ | well-fed | normal | 5 |
| Pgpa-4-pkg-1gf_fasted | *adEx2228[gpa-4p::egl-4CA rol-6p::GFP]* | fasted/refed | normal | 5 |
| Pgpa-4-pkg-1gf_unfasted | *adEx2228[gpa-4p::egl-4CA rol-6p::GFP]* | well-fed | normal | 13 |
| pkg-1_fasted | *egl-4(ks62) IV* | fasted/refed | normal | 6 |
| pkg-1_fasted_azt | *egl-4(ks62) IV* | fasted/refed | Azt | 2 |
| pkg-1_fasted_mix | *egl-4(ks62) IV* | fasted/refed | Mix | 3 |
| pkg-1gf_ASI-_fasted | *egl-4(ad450sd) IV; Is[gpa-4p::recCaspase gcy-27p::recCaspase gcy-27p::gfp]*^2^ | fasted/refed | normal | 5 |
| pkg-1gf_ASI-_unfasted | *egl-4(ad450sd) IV; Is[gpa-4p::recCaspase gcy-27p::recCaspase gcy-27p::gfp]*^2^ | well-fed | normal | 7 |
| pkg-1gf_fasted | *egl-4(ad450sd) IV* | fasted/refed | normal | 6 |
| pkg-1gf_unfasted | *egl-4(ad450sd) IV* | well-fed | normal | 8 |
| pkg-1_IS500_fasted | *egl-4(ks62) IV; pyIs500[odr-3p::gfp::egl-4]^3^* | fasted/refed | normal | 6 |
| pkg-1_IS500_unfasted | *egl-4(ks62) IV; pyIs500[odr-3p::gfp::egl-4]^3^* | well-fed | normal | 5 |
| pkg-1-Pgpa-4-pkg-1_fasted | *egl-4(ks62) IV; adEx2233[gpa-4p::egl-4 rol-6::GFP]* | fasted/refed | normal | 5 |
| pkg-1-Pgpa-4-pkg-1gf_fasted | *egl-4(ks62) IV; adEx2236[gpa-4p::egl-4CA rol-6::GFP]* | fasted/refed | normal | 5 |
| pkg-1-Pgpa-4-pkg-1gf_unfasted | *egl-4(ks62) IV; adEx2236[gpa-4p::egl-4CA rol-6::GFP]* | well-fed | normal | 12 |
| pkg-1-Pgpa-4-pkg-1_unfasted | *egl-4(ks62) IV; adEx2233[gpa-4p::egl-4 rol-6::GFP]* | well-fed | normal | 8 |
| pkg-1_Posm-10-pkg-1_fasted | *egl-4(n479) IV; udEx95(elt-2p::gfp; osm-10p::gfp::egl-4)*^4^ | fasted/refed | normal | 1 |
| pkg-1_Posm-10-pkg-1_unfasted | *egl-4(n479) IV; udEx95(elt-2p::gfp; osm-10p::gfp::egl-4)*^4^ | well fed | normal | 3 |
| pkg-1_Ppkg-1-pkg-1_fasted | *egl-4(ks62) IV; adEx2146[egl-4p::egl-4 rol-6::GFP]* | fasted/refed | normal | 5 |
| pkg-1_Ppkg-1-pkg-1_unfasted | *egl-4(ks62) IV; adEx2146[egl-4p::egl-4 rol-6::GFP]* | well-fed | normal | 18 |
| pkg-1_Ptax-4-pkg-1_fasted | *egl-4(ks62) IV; adEx2143[tax-4::egl-4 rol-6::GFP]* | fasted/refed | normal | 4 |
| pkg-1_Ptax-4-pkg-1_unfasted | *egl-4(ks62) IV; adEx2143[tax-4::egl-4 rol-6::GFP]* | well-fed | normal | 14 |
| pkg-1_unfasted | *egl-4(ks62) IV* | well-fed | normal | 12 |
| pkg-1_unfasted_azt | *egl-4(ks62) IV* | well-fed | Azt | 9 |
| pkg-1_unfasted_mix | *egl-4(ks62) IV* | well-fed | Mix | 6 |
| Ptax-4-pkg-1gf_fasted | *adEx2225[tax-4p::egl-4CA rol-6p::GFP]* | fasted/refed | normal | 3 |
| Ptax-4-pkg-1gf_unfasted | *adEx2225[tax-4p::egl-4CA rol-6p::GFP]* | well-fed | normal | 7 |
| tax-4_unfasted | *tax-4(p678) III* | well-fed | normal | 6 |
| ttx-3_unfasted | *ttx-3(ot22) X* | well-fed | normal | 12 |
| wt_fasted | *+* | fasted/refed | normal | 14 |
| wt_fasted_azt | *+* | fasted/refed | Azt | 4 |
| wt_fasted_EtOH | *+* | fasted/refed | EtOH | 6 |
| wt_fasted_mix | *+* | fasted/refed | Mix | 5 |
| wt_unfasted | *+* | well-fed | normal | 8 |
| wt_unfasted_azt | *+* | well-fed | Azt | 12 |
| wt_unfasted_mix | *+* | well-fed | Mix | 21 |
| wt_unfasted_mix_EtOH | *+* | well-fed | Mix, EtOH | 4 |
| wt_unfasted_OA | *+* | well-fed | OA | 6 |
| **Total** |  |  |  | **363** |

^1^Normal: *E coli* HB101. Azt: *E coli* HB101 treated with 5 μg/ml aztreonam [1]. Mix: 1:9 mixture of aztreonam-treated and untreated *E coli* HB101. EtOH: medium contained 0.1% ethanol. (This is a vehicle control for OA.) OA: medium contained 600 mM oleic acid + 0.1% ethanol.

^2^PY7504, similar to PY7505 of Beverly et al. [2].

^3^Lee et al. [3].

^4^This strain was a gift from Denise Ferkey.

References

1. Ben Arous J, Laffont S, Chatenay D (2009) Molecular and sensory basis of a food related two-state behavior in C. elegans. PLoS One 4: e7584.

2. Beverly M, Anbil S, Sengupta P (2011) Degeneracy and neuromodulation among thermosensory neurons contribute to robust thermosensory behaviors in Caenorhabditis elegans. J Neurosci 31: 11718-11727.

3. Lee JI, O'Halloran DM, Eastham-Anderson J, Juang BT, Kaye JA, et al. (2010) Nuclear entry of a cGMP-dependent kinase converts transient into long-lasting olfactory adaptation. Proc Natl Acad Sci U S A 107: 6016-6021.
